# Drop-In Capability of Solketal in Diesel and Gasoline Fuels Containing Biodiesel, HVO, Fossil Diesel, and Gasoline Based on Standard Physical and Chemical Fuel Properties

**DOI:** 10.1021/acsomega.5c12056

**Published:** 2026-04-16

**Authors:** Julian Türck, Fabian Schmitt, Sumit Agarwal, Jens Utecht, Ralf Türck, Wolfgang Ruck, Jürgen Krahl

**Affiliations:** † 414954 Leuphana University Lüneburg, School of Sustainability, Universitätsallee 1, Lüneburg 21335, Germany; ‡ Tecosol GmbH, Jahnstraße 2, Ochsenfurt 97199, Germany; § Department of Physical Chemistry, 39428 Physikalisch-Technische Bundesanstalt, Bundesallee 100, Braunschweig 38116, Germany; ∥ L.M.U. Business Consulting GmbH, Im Baumgarten 22, Obrigheim 67283, Germany; ⊥ Fuels Joint Research Group, www.fuels-jrg.de, Masch 16, Röttgesbüttel 38531, Germany; # OWL University of Applied Sciences and Arts, Campusallee 12, Lemgo 32657, Germany

## Abstract

This study evaluates the drop-in capability of solketal in terms of the physical and chemical parameters defined for diesel and gasoline fuels. To this end, miscibility gaps revealed that solketal is immiscible with pure hydrocarbons and lower aromatic fossil diesel fuel (18.84 wt %), while biodiesel acts as an effective solubilizer due to its amphiphilic structure. Solketal exhibited a significant influence on fuel polarity, as demonstrated by increases in permittivity (ε) and decreases in interfacial tension (it) (in binary blends with biodiesel: 15.7% (ε) and 43.6% (it); in ternary alkane-biodiesel blends: 9.8% (ε) and 68.3% (it)). At 3 vol % solketal, Diesel R33 remained EN 590 compliant despite increased density and reduced ignition quality and flash point. Density increased by ∼0.9% per 5 vol % solketal in binary biodiesel blends and by 0.6–1.0% in ternary hydrotreated vegetable oil blends. At maximum solketal contents (20 vol % binary, 9 vol % ternary), cetane number decreased by up to 15% and 5.7%, respectively, while cetane index decreased by up to 60.7% in binary blends. The flash point decreased by up to 41% in binary and 2–6% in ternary blends. Pure solketal exhibited a vapor pressure 32–50 times lower than ethanol and methanol at 100 °C but increased biodiesel vapor pressure by a factor of 1.07 at 20 vol % blending, indicating nonideal behavior. In gasoline blends, vapor pressure decreased by only 8–11%, maintaining acceptable Reid vapor pressure.

## Graphical abstract

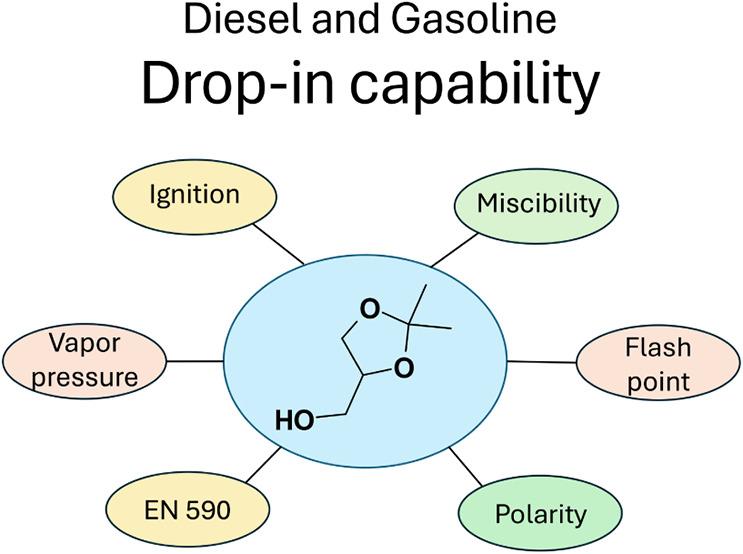

## Introduction

The integration of novel renewable fuels into existing vehicle fleets represents a promising pathway for short-term mitigation of greenhouse gas emissions. This attribute, commonly referred to as drop-in capability, encompasses several critical dimensions, with engine compatibility being of primary importance. This includes the extent to which the fuel is compatible with modern injection systems,^1^ sealing materials, engine oil, and exhaust aftertreatment technologies such as diesel particulate filters, oxidation catalysts, and selective catalytic reduction (SCR) catalysts.^2^ The initial stage of assessment involves examining the effects of the new fuel on its physical and chemical properties and on key standard parameters. Deviations in these properties may lead to diminished engine efficiency or, in severe cases, render the fuel unsuitable for practical application. Moreover, such variations can introduce additional challenges for commercialization, both in terms of regulatory compliance and technical feasibility.

The need to reduce emissions has accelerated the search for new drop-in components. New solutions were being sought, particularly in the diesel fuel sector. One example of this is Diesel R33, which contains 33% renewable components. This is composed of 7% biodiesel and 26% hydrotreated vegetable oils (HVO).^3^ However, HVO reduces the density, so that the lower limit of the Diesel Standard EN590 is reached at some point.^4,5^ This is due to the chemical composition, which is free of aromatics compared to fossil diesel fuel (DF). However, the absence of aromatics can result in a soot reduced combustion.^6,7^ HVO was the first hydrocarbon mixture to be introduced to the market that has the same boiling range as DF. In addition, the isomerization step makes it possible to adjust the cold properties to such an extent that it can be used as biokerosene.^8^

In the future, the aim is to develop and introduce further sources and possibilities for producing renewable alkanes. Examples of this are e-fuels and Fischer–Tropsch alkanes, which can be produced via carbon capture technologies.^9,10^ Of particular interest is the search for polar drop-in components that have a higher molecular oxygen density. Oxygenated fuels have the potential to combust with significantly reduced soot formation.^11^ A representative example is polyoxymethylene ether (OME), which can be synthesized from methanol and formaldehyde. Due to the absence of carbon–carbon bonds, OME undergoes nearly soot-free combustion.^12,13^ The presence of additional oxygen-containing functional groups increases the overall polarity of the fuel, thereby influencing its drop-in compatibility.^14^ This may lead to the formation of miscibility gaps, while the enhanced solubility of metals can adversely affect exhaust aftertreatment systems.^15^ Furthermore, the efficiency of water separation devices may be impaired.^16^ Polarity can influence the nature and strength of intermolecular interactions. Another relevant indicator of such effects is the vapor pressure behavior. Furthermore, Raoult’s law and the corresponding activity coefficients allow conclusions to be drawn regarding the ideality of the mixture. In general, the vapor pressure is of relevance for the air–fuel mixture formation during the injection and ignition processes.^17^

Another promising raw material for the production of a polar drop-in component is glycerin. Glycerin is a byproduct of biodiesel production and can be used in both the pharmaceutical and food markets.^18^ The glycerin world market increased since 30 years with a rate of 150,000 t/a and an expected quantity of 6,000,000.^19^ As a pure substance, glycerin cannot be used as a fuel component because it emits acrolein,^20^ has a lower heating value,^21^ is corrosive^22^ and is too polar to be miscible with diesel alkanes.^23^ One way to make glycerin usable as a fuel is through chemical derivatization. A promising option is conversion to solketal.^24^ Solketal has similar fuel properties to ethanol (reduction in gum, increase in octane number^25^), which qualifies it as a gasoline fuel. Fuels containing solketal were tested on a gasoline engine test bench to determine their emission profile. It was found that hydrocarbon and CO emissions decrease, while CO, NO_X_ and brake-specific fuel consumption increase.^26,27^ However, investigations were also carried out on diesel fuel. Solketal nanoemulsions were investigated in a diesel engine,^28,29^ as well as the influence of solketal on spray formation during injection.^30^ From the perspective of ignition properties as a diesel fuel, it tends to reduce the cetane number.^31^ However, it also exhibits interesting diesel fuel properties, as it slows down the oligomerization process.^32,33^ In addition, it shows synergy with antioxidants, which results in increased oxidation stability.^34^ The precipitation of polar aging products in the course of fuel aging can be reversibly dissolved with alcohols.^35^ In general, solketal is produced by means of a proton-catalyzed condensation reaction (see Figure 1).

**Figure 1:**
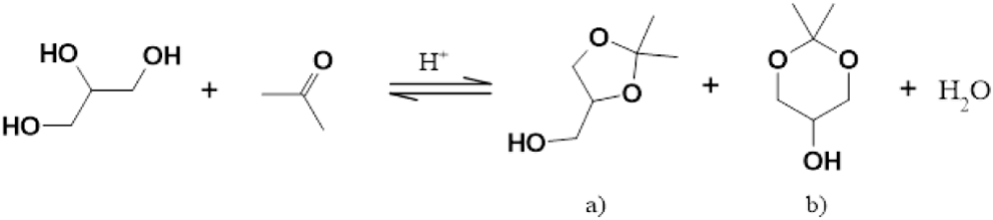
Proton-catalyzed condensation reaction for the synthesis of solketal. Two constitutional isomers are formed: a) dioxolane derivative and b) dioxane derivative.

The reaction proceeds via an acid-catalyzed ketalization, in which glycerin reacts with acetone to form solketal and water. Various synthetic routes can be employed for the production of solketal depending on the proton source. The reaction itself is exothermic and therefore requires careful temperature control.^36–38^ In addition, catalyst-free reactions using supercritical acetone are conceivable.^39^ During solketal synthesis two constitutional isomers are formed, a five-membered dioxolane derivative and a six-membered dioxane derivative (see Figure 1). The five-membered dioxolane derivative predominates, while the six-membered dioxane derivative is produced only in trace amounts (approximately 99:1).^24^

The present study investigates the influence of solketal on the physical and chemical properties of diesel fuel in accordance with the EN 590 specification. Particular emphasis is placed on the effect of solketal on fuel polarity, determined through measurements of dielectric permittivity as well as interfacial and surface tension. In addition, the ignition characteristics, flash point, and vapor pressure behavior are systematically evaluated.

## Experimental Section

### Used Fuels and Chemicals

The analytical data for the fuels employed in this study are provided in the Supporting Information. In general, different fuels were used for the respective experimental investigations. An important prerequisite was that the influence of water was kept to a minimum, as it affects the physical and chemical properties. Table 1 lists the corresponding table numbers from the Supporting Information that present the relevant analyses, along with details of the fuel suppliers. This procedure was chosen because fresh fuel was used for each test. As a result, 11 different fuels were used. The fuels were primarily obtained from ASG-Analytik-Service GmbH (Germany) and Louis Dreyfus B.V. (Netherlands). The biodiesel utilized was produced from rapeseed oil. An exception is the vapor pressure analysis, which was conducted using fossil gasoline (OF) purchased from ASG. All chemicals used were of analytical grade and commercially sourced. Solketal (purity ≥98%) was obtained from Merck KGaA (Germany), ethanol (≥99%) from Carl Roth GmbH (Germany), and methanol (≥99%) from Tecosol GmbH (Germany).

**Table 1:** Overview of the Table Numbers in the Supporting Information That Present the Fuel Analyses Corresponding to the Respective Experimental Investigations

Investigation	Biodiesel	HVO	DF
Miscibility gap	S2	S3	S4
EN590	S5	S6	S7
Solketal influence	S8	S9	S10
Vapor pressure	S11	/	/

### Investigated Blends

In this study, various fuels and fuel compositions were examined depending on the respective experimental objective. In order to evaluate miscibility gaps, binary blends are composed of diesel fuel, either DF or HVO and biodiesel. Solketal is subsequently added as a third component to these blends to assess its influence on miscibility and the evaluated fuel properties. The purpose of this was to evaluate the identified aromatic influence and in greater depth. Based on the blending results, the influence of solketal were used to evaluate miscibility related polarity, both binary and ternary blend systems were selected. The binary system consisted of biodiesel–solketal mixtures (BxSy), whereas the ternary system comprised HVO, biodiesel, and solketal, maintaining a constant biodiesel content of 15 wt % (HVOxB15Sy). The 15 wt % was derived from the ternary mixture diagram of HVO, biodiesel, and solketal. The objective was to use a relatively uncomplex binary fuel that would allow for a more simplified evaluation of the direct influence of solketal. The system then became more complex with the addition of HVO. In order to limit the biodiesel parameter and influence, the biodiesel content was set at 15 vol %. The influence of solketal on physical and chemical parameters was qualitatively evaluated within a Diesel R33 matrix (with and without 3 wt % solketal). Diesel R33 consists of 33% renewable share (26 vol % HVO and 7 vol % biodiesel). This fuel matrix was chosen because it has the highest proportion of renewable fuels that still complies with the EN590 standard. In order to investigate the influence of solketal on the identified parameters of EN590, the same fuel systems were used as in the polarity analysis. After the transition between the liquid and gas phases had been identified as an influenced physical property, vapor pressure investigations were carried out. In this study, solketal was compared as a pure component with chemically similar established fuels such as methanol and ethanol (includes 50:50 mixtures of solketal with methanol or ethanol). Furthermore, BxSy blends, and OF containing 10 and 20 vol % solketal were analyzed regarding their vapor pressure characteristics. Both fuel systems were investigated, as biodiesel has a lower vapor pressure, which can lead to increased engine oil dilution, whereas vapor pressure is a crucial fuel property for OF. The percentage units used were selected based on the investigations. Depending on the device, either vol % or wt % was preferred.

### Determination of the Miscibility Gap

Ternary blend diagrams were generated to determine the miscibility gap of solketal. The objective was to examine the miscibility gaps depending on the chemical compositions. Therefore, the temperature was kept constant at room temperature. Moreover, the influence of water was neglected. The blends were prepared volumetrically (vol %) in defined increments. For each preparation series, the total blend volume was adjusted to 1 mL and transferred into test tubes. After homogenization, the blends were examined for phase separation. The evaluation was conducted by visual inspection under standardized lighting and ambient conditions. The resulting data were plotted in ternary mixture diagrams, indicating the single-phase and two-phase regions of the respective blends. Initially, all components were blended in 10 vol % steps ranging from 0 to 100%. Building on this, an additional ternary diagram was constructed to illustrate the region of the miscibility gap. On this basis, the boundaries of the miscibility gap were determined with a resolution of 1 vol %.

### Measurement of the Polarity

Dielectric relaxation spectroscopy (permittivity ε) was performed using a Flucon Epsilon+ device. The permittivity was measured at a frequency of 100 kHz (at room temperature). The sample volume was 8 mL. When charge carriers align with the vector of an electric field, a polarization field is generated. Therefore, permittivity and polarization are interdependent. Table 2 shows the permittivity (100 kHz at 25 °C) of the pure components.

**Table 2:** Permittivity of the Fuels Used at 100 kHz and 25 °C

	Solketal	Biodiesel	HVO	DF
ε_r_ (100 kHz) at 25 °C	9.65	3.45	2.06	2.18

Surface and interfacial tensions were determined at ASG Analytik Service GmbH (Germany). The measurements were carried out at a temperature of 25 °C, using a sample volume of 50 mL. Surface tension describes the physical phenomenon in which liquids tend to minimize their surface area due to cohesive intermolecular forces. These forces act at the liquid interfaces (between liquid and gas phase) and are directed outward. Interfacial tension was determined analogously to surface tension using the same analytical device. The same measurement parameters and sample volumes were applied. Interfacial tension describes the molecular interactions between two immiscible liquids. The stronger molecules are bonded within one phase and the weaker their interactions with the second phase, the greater the interfacial tension.

### Determination of the EN 590

In order to characterize and determine the influence of solketal on the physical and chemical properties, the Diesel R33 blends were tested in accordance with the EN 590 diesel fuel standard. The parameters specified in EN590 were measured both externally at ASG Analytik Service GmbH (Germany) and internally. An overview of which measurements were conducted internally or externally is provided in the Supporting Information. The parameters measured internally are summarized in Table 3, which also lists the sample quantities and the instruments used. Parameters that are examined in greater detail in the discussion section are described individually.

**Table 3:** Presentation and Summary of the EN 590 Parameters, Including the Respective Measurement Instruments, Analytical Methods, and Sample Quantities Which Were Measured Internally

EN590 Parameter	Device	Method	Sample quantity
Density (15 °C)	Anton Paar DMA 4100M	Oscillating U-tube	3–5 mL
Sulfur content	TE Instruments Xplorer-NS	UV-Fluorescence	30 μL
Water content	Metrohm KF 756	Coulometric Titration	1–2 g
Total pollution	Cellulose nitrate filter	Gravimetric	300 mL
Kin. Viscosity (40 °C)	Anton Paar SVM 3001	Stabinger	5 mL
Cold Filter Plugging Point	PAC OptiFPP	Temperature increments	45 mL

### Determination of Cetane Number (CN), Cetane Index (CI), and Flash Point

The cetane number (CN) and cetane index (CI) were determined by ASG Analytik-Service GmbH (Germany). The CN serves as a measure of the ignition quality of diesel fuels and was determined in accordance with EN 17155:2018 using a standardized test procedure based on a single-cylinder rapid compression engine. The CI represents a calculated approximation of the cetane number and was determined in accordance with EN ISO 4264:2018. The calculation is based on selected physical properties of the diesel fuel, particularly the density at 15 °C and characteristic distillation temperatures corresponding to 10% (*T*_10_), 50% (*T*_50_), and 90% (*T*_90_) of the evaporated volume.

The flash point was determined in accordance with EN ISO 2719:2016 using an Anton Paar PMA 5 apparatus, employing the Pensky–Martens closed-cup method. In this procedure, a defined fuel volume of 60 mL is gradually heated in a sealed test cup. At specified temperature intervals, a small test flame is introduced through an opening to initialize ignition. For flash points below 110 °C, the test flame is applied in 1 °C increments (Method A), while for samples exceeding 110 °C, 2 °C increments are used (Method B).

### Determination of Vapor Pressure

The vapor pressure curves of solketal and solketal-containing fuels were determined using a Herzog HVP 972 vapor pressure tester. For each measurement, a defined fuel volume of 10 mL was introduced into a gastight, sealed test cell and subsequently heated in a thermostatic heating block. The resulting vapor pressure curve represents the pressure as a function of temperature. From these data, characteristic parameters such as the Reid vapor pressure (RVP), initial boiling point, and evaporation behavior can be derived. The RVP is normatively defined as the absolute vapor pressure of a liquid measured at 37.8 °C.

## Results and Discussion

### Investigation of Miscibility Gaps in Ternary Blends

In order to conduct fuel-specific investigations, it is essential to first verify the stability of the respective blends. In general, it was observed that more solketal dissolved as the temperature rises, while less dissolved as the temperature decreased. Solketal was found to be immiscible with pure hydrocarbons such as HVO. In the case of DF, miscibility is primarily governed by the aromatic content, as aromatic compounds can act as solubilizing agents. The low aromatic content of the DF used in this study (18.84 wt %) resulted in immiscibility with solketal. However, diesel fuels with higher aromatic contents, such as 24.4 wt %, were found to achieve miscibility with up to 3 vol % solketal.^30^ To obtain stable blends, the addition of a third amphiphilic component is therefore required. Biodiesel exhibits such amphiphilic properties due to its polar ester functional group and nonpolar hydrocarbon chain.^40^ In general, biodiesel and solketal are completely miscible. The following section examines the miscibility gaps of solketal in ternary mixtures with biodiesel and HVO or DF. The corresponding ternary mixture diagrams, which provide a complete overview of the compositional range (0–100 vol %), are presented in the Supporting Information.

The results of the analysis indicated that the blending limit for DF ranged between 20 and 30 vol % biodiesel. At DF contents below 30 vol %, up to 80 vol % solketal could be incorporated due to a polarity transition from no polar to polar at high biodiesel/solketal ratios. Since single-phase mixtures were already observed at a biodiesel content of 20 vol %, the ternary mixture diagram was constructed up to 20 vol % biodiesel in increments of 2 vol % (see Figure 2a). This allowed the miscibility limit to be further refined, establishing a critical range between 8 and 10 vol % biodiesel. Within this range, blends were subsequently analyzed in 1 vol % increments. It was determined that, for this DF, the investigated miscibility gap began at 9 vol % biodiesel and 1 vol % solketal, and extended up to approximately 7 vol % solketal at a biodiesel content of 14 vol %.

**Figure 2:**
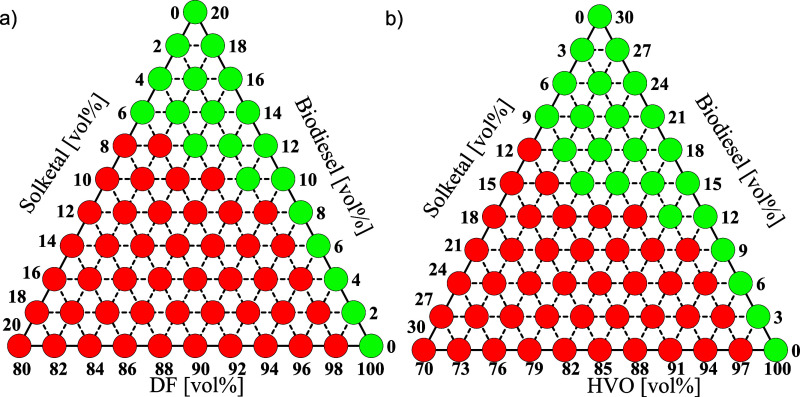
Ternary mixture diagram of a) DF, biodiesel, and solketal and b) HVO, biodiesel, and solketal. The mixing range for a) was between 0 and 20 vol % for biodiesel and solketal and between 80 and 100 vol % for DF (in 2 vol % increments). For b): the mixing range was between 0 and 30 vol % for biodiesel and solketal and between 70 and 100 vol % for HVO (in 3 vol % increments). The green markers represent stable single-phase blends, while the red markers indicate unstable two-phase blends.

For HVO-based blends, the absence of aromatic compounds led to a shift in the miscibility limit toward higher biodiesel contents (see Figure 2b). Stable blends were obtained from 30 vol % biodiesel onward, with single-phase mixtures observed above 40 vol %. Blends containing low HVO fractions also exhibited stable solutions at a biodiesel content of 20 vol %. To identify the miscibility range in more detail, the corresponding ternary mixture diagram was generated up to 30 vol % biodiesel in increments of 3 vol %. It was observed that up to 9 vol % solketal was miscible at 21 vol % biodiesel, while only 1 vol % solketal was miscible at 11 vol % biodiesel.

In summary, it can be said that the hydrocarbon-based diesel fuels used are decisive in determining the extent to which the blend gap shifts. An increased aromatic content enhances the polarizability and thus the dielectric response of the hydrocarbon matrix.^41^ As a result, dipole–induced dipole interactions and π-associated interactions are promoted, improving the compatibility and miscibility with polar oxygenates such as solketal.^42–44^ The higher the aromatic content, the more “polar” the hydrocarbon mixture is. In order to ensure drop-in capability in existing systems such as existing fleets or blending stations, the blends should be based on the miscibility gaps in HVO. Regardless of this, it is important to understand the extent to which solketal influences the polarity of the overall mixture. Depending on how much solketal is added, it may be necessary to adjust the correct aromatic content.

### Influence of Solketal on Polarity of the Fuel

The polarity of a fuel cannot be measured directly. However, correlations between permittivity as well as surface and interfacial tension with varying solketal content allow indirect conclusions to be drawn regarding the polarity and its changes. Türck et al. have previously investigated the influence of solketal on permittivity and surface tension.^31^ Their results indicated that both parameters tend to increase with rising solketal concentration. However, those studies examined ternary mixtures containing DF while simultaneously addressing the influence of an antioxidant. Furthermore, the biodiesel content was varied, exerting a stronger effect. To enable a more precise analysis, the present study employed a simplified system. Initially, the influence of solketal was investigated in a binary biodiesel–solketal system (BxSy), followed by an extension to a ternary system comprising HVO, biodiesel, and solketal at a constant biodiesel content (HVOxB15Sy). The corresponding results for permittivity and interfacial tension are presented in Figures 3 and 4.

**Figure 3:**
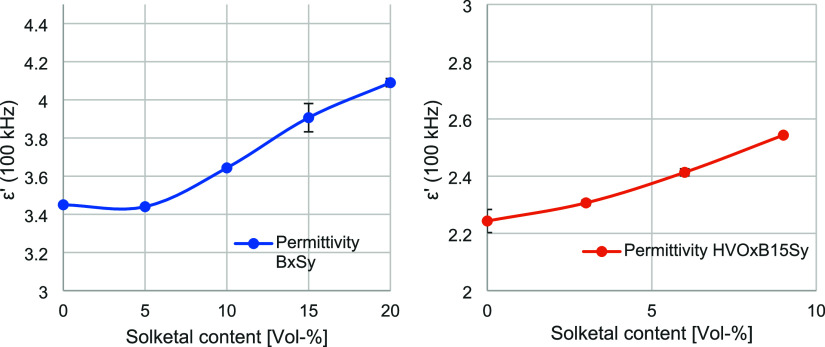
Relative permittivity ε_r_ (100 kHz) at room temperature for varying solketal contents for the binary system BxSy (left, blue) and the ternary system HVOxB15Sy (right, orange) (number of measurements *n* = 3).

**Figure 4:**
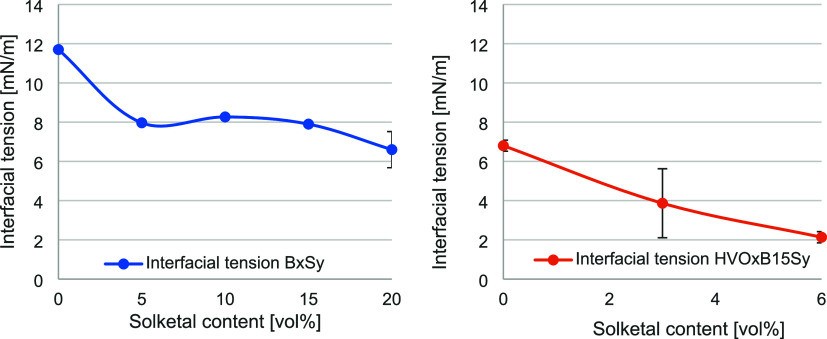
Interfacial tension at room temperature for varying solketal contents for the binary system BxSy (left, blue) and the ternary system HVOxB15Sy (right, orange) (*n* = 3).

In addition, the surface tension was measured for the same blend compositions. No significant changes were observed. The BxSy system exhibited values ranging from 31.2 to 31.4 mN/m, while the HVOxB15Sy system ranged from 27.1 to 27.3 mN/m. These findings are consistent with the observations of Türck et al., indicating that the primary influence on surface tension arises from the biodiesel content. This effect may be attributed to the surfactant-like molecular structure of biodiesel due to its amphiphilic properties.^45^ Interestingly, variations in the concentration of the fuel components within both systems did not lead to measurable changes in surface tension. In contrast, a distinct behavior was observed for the interfacial tension. The addition of solketal resulted in a pronounced reduction in both systems. In the biodiesel system, a decrease of 43.6% was observed in blends containing up to 20 vol % solketal, whereas in the ternary HVO system, the reduction reached 68.3% at solketal concentrations up to 6 vol %. Notably, in both systems without solketal, pure biodiesel exhibited a higher interfacial tension than the biodiesel–HVO mixture, with an increase of 41.9% relative to the latter.

The distinction between surface tension and interfacial tension arises from the different states of matter involved. While interfacial tension describes the tension at the interface between two immiscible liquid phase, surface tension refers to the tension at the interface between a liquid and its gaseous phase. The observed reduction in interfacial tension indicates enhanced miscibility between the polar and nonpolar phases, reflecting an increase in overall polarity induced by the addition of solketal. This interpretation is supported by the permittivity measurements, which revealed an increase of up to 15.7% for the BxSy system and 9.8% for the HVOxB15Sy system upon solketal addition. Furthermore, the BxSy system exhibited higher permittivity values overall, attributable to the inherently higher permittivity of biodiesel compared to HVO.^31^

### Identification of the Affected EN 590 Parameters by Solketal

The increase in polarity can have an influence on the physical and chemical parameters of the EN590. To determine the parameters, solketal was blended with EN590-compliant Diesel R33 in a concentration of 3 vol %. Table 4 shows the relevant parameters.

**Table 4:** Comparison of the Physical and Chemical Fuel Properties Specified in the EN 590 between Diesel R33 and Diesel R33 + 3 vol % Solketal (S)^a^

Parameter	R33 + 3 vol % S	R33	Unit	Min.	Max.
CN	64.8	65.8	/	51.0	/
CI	62.8	65.8	/	46.0	/
Density (15 °C)	820.3	815.2	kg/m^3^	820	845
Polycyclic aromatic hydrocarbons	3.6	3.7	% (w/w)	/	8.0
Sulfur content	<5 (<1)	<5 (<1)	mg/kg	/	10
Flash point	85.5	96.0	°C	>55	/
Coke residue (10% D.)	<0.10	<0.1	% (w/w)	/	0.3
Ash content (775 °C)	<0.001	<0,001	% (w/w)	/	0.01
Water content	70	<30	mg/kg	/	200
Total pollution	<12	<12	mg/kg	/	24
Corrosive effect on copper	1	1	Corr. Deg.	/	1
Oxidation stability	<1	2	g/m^3^	/	25
Oxidation stability	29.6	34.4	min	60	/
HFRR (lubricity at 60 °C)	170	180	μm	/	460
Kinematic viscosity (40 °C)	2.705	2.736	mm^2^/s	2.000	4.500
Volume at 250 °C	16.7	14.2	% (V/V)	/	<65
Volume at 350 °C	98.7	98.3	% (V/V)	85	/
95% point	339.2	340.3	°C	-	360
CFPP	–18	–18	°C	-	^b^
Manganese (Mn)	<0.5	<0,5	mg/L	/	2.0

a The table further displays the applicable limits of the standard.

b 15.04–30.09 max 0 °C; 01.10–15.11 max −10 °C; 16.11–28.03 max −20 °C; 01.03–14.04 max −10 °C.

In principle, the table illustrated that blending 3 wt % solketal into Diesel R33 results in all parameters complying with the EN 590 standard. It has been demonstrated that solketal influences the ignition characteristics of diesel fuel. In the literature, solketal is described as a component that increases the octane number.^25^ Components that enhance knock resistance generally exhibit lower autoignition tendencies,^46^ as engine knocking is partly attributable to premature autoignition events.^47^ Consequently, a reduction in the cetane index was observed. Moreover, solketal caused a pronounced increase in fuel density even at an addition level of 3 vol %. This effect can be attributed to the comparatively high intrinsic density of solketal (1,063 kg/m^3^). With increasing solketal concentration, the density increased linearly, a correlation also reported in other studies.^48^ In the binary mixtures (BxSy), each solketal increment corresponded to an approximate 0.9% increase in density, whereas the ternary mixtures (HVOxB15Sy) exhibited lower linearity, with variations between 0.6 and 1%. The observed increase in density suggests that solketal promotes stronger intermolecular interactions, potentially associated with reduced molecular distances. However, this effect could not be clearly correlated with kinematic viscosity. Although solketal, as a pure substance, exhibits a higher intrinsic viscosity than the other fuel components,^49^ no significant change in overall viscosity was observed in either the binary or ternary blends investigated.

The observed reduction in lubricity was determined to be a statistical deviation, as subsequent measurements did not reveal a systematic trend. Both standard parameters of oxidation stability were affected by the presence of solketal. In general, the induction period reflects the increase in electrical conductivity resulting from decomposition reactions. The corresponding decomposition products possess boiling points below 110 °C and condense in the eluate. The present study did not further address this aspect, as the influence of solketal on fuel aging has been comprehensively discussed in the literature. Türck et al. reported that solketal reduced the extent of fuel aging, as a higher fraction of unaged biodiesel was detected by size exclusion chromatography.^33^ Moreover, solketal was found to react with formed epoxides, thereby slowing the progression of oxidative aging.^32^ In addition, Kerkel et al. demonstrated that solketal enhances the effectiveness of antioxidants, leading to improved oxidation stability.^34^ This effect, however, is not apparent in Table 5, since the fuel components used were additive-free. The interaction is also related to solketal’s potential corrosive effect on copper, as the acid number may increase during the aging of solketal-containing fuels.^50^ No deviation from the R33 reference fuel was observed in this regard.

**Table 5:** Determination of the Deviation and the Activity Coefficient (γ) for the Two Mixtures: Solketal–Methanol and Solketal–Ethanol

Mixture	*p*_theor_ at 30 °C [kPa]	*p*_exp_ at 30 °C [kPa]	*p*_deviation_ [%]	Activity coefficient γ
MeOH:solketal (50:50)	16.82	17.83 ± 0.09	6	1.06
EtOH:solketal (50:50)	7.37	8.37 ± 0.05	14	1.14

No significant influence on the cold and distillation properties was found. In contrast, a significant change in the flash point was measured. There were no solketal-related impurities in the chemical composition of polyaromatics, sulfur and manganese. The same applied to the total pollution, ash and coke content. In contrast, higher water absorption was observed in the water content. Since solketal is completely water-soluble due to its polarity as a glycerol derivative, solketal became a solubilizer for higher water contents. Such behavior had also been observed in 1-octanol fuel investigations.^51^

### Analysis of CN, CI, and Flash Point

Following the identification of the affected EN 590 parameters, this section systematically investigates the influence of solketal. Among the most critical parameters for diesel fuels is the ignition behavior, characterized by the CN and the CI. As a pure compound, solketal exhibited no ignition behavior under diesel engine conditions, highlighting its gasoline-like characteristics. In this study, solketal was therefore examined within binary and ternary mixtures containing renewable fuel components (see Figure 5). The nomenclature applied follows the convention described in the experimental section, with binary systems denoted as BxSy and ternary systems as HVOxB15Sy.

**Figure 5:**
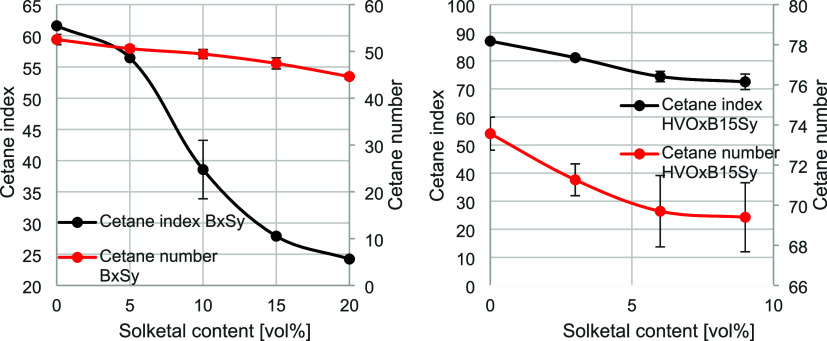
Trends of the cetane number (red) and cetane index (black) with increasing solketal content for the binary system BxSy (left) and the ternary system HVOxB15Sy (right) (*n* = 3).

The trend of decreasing CN with increasing solketal content was confirmed. The reduction from pure biodiesel (B100) to B80S20 was approximately 15%. Overall, the decrease in CN per 5 vol % solketal addition ranged between 2 and 6%, exhibiting a relatively linear relationship. Notably, the binary mixtures remained combustible under diesel engine conditions up to a solketal content of 20 vol %. This effect was even more pronounced in the theoretically determined CI, which showed a sharper decline of 60.7% between B100 and B80S20. The greater reduction may be attributed to the pronounced influence of solketal’s density. Furthermore, this may indicate that solketal exerts a stronger effect on the boiling behavior of the blend. A larger deviation was observed for B90S10, which significantly affected the curve progression; however, when this error is considered, the overall trend remains steeper. Between B100 and B95S5, the CI decreased by approximately 9%, followed by more substantial reductions of 32% and 28%, before eventually leveling off with a final decrease of 13%.

In the ternary blends, the trend of decreasing ignitability with increasing solketal content was likewise observed. The total reduction in the ternary system, determined by comparing HVOB15S0 and HVOB15S9, amounted to 5.7%. Since a lower solketal fraction was used in the ternary mixtures, the decrease between the two systems was compared with B90S10, which contained one vol % more solketal. In this case, a 5.9% reduction in CN compared to B100 was observed. This suggests that the influence of solketal on ignition behavior is proportional, irrespective of the fuel matrix. Overall, both CN and CI values were higher in the ternary blends than in the binary mixtures. Here, HVO acted as a cetane number enhancer due to its high inherent ignitability as a pure component (see fuel analysis in the Supporting Information). Without solketal addition, CN and CI were approximately 40–41% higher than in the corresponding binary systems. The trends of CN and CI in the ternary blends were more consistent than in the binary mixtures. However, as observed in the binary systems, a more pronounced decrease was evident for the CI. Furthermore, it was noted that measurement uncertainty increased with rising solketal content and was generally larger, except for B90S10, indicating that the additional component introduced more complex interactions affecting ignition-related parameters such as liquid–gas phase transitions and density. With regard to EN590 drop-in capability, both the cetane number and cetane index of the binary blend showed that the lower limit between 5 and 10% was exceeded. For ternary blends, the addition of HVO ensured that all blends were within the standard.

As discussed, solketal addition caused a linear increase in total density. The liquid–gas phase transitions suggest altered flash point behavior, since both density and evaporation characteristics influence the CI. Therefore, subsequent analysis focused on the flash point. Independently of the liquid–gas phase behavior, the flash point is a safety-relevant parameter. Due to the requirements of the EN590 standard, according to which the fuel must have a flash point of >55 °C, all blends were verified as being directly usable. Figure 6 illustrates the corresponding progressions.

**Figure 6:**
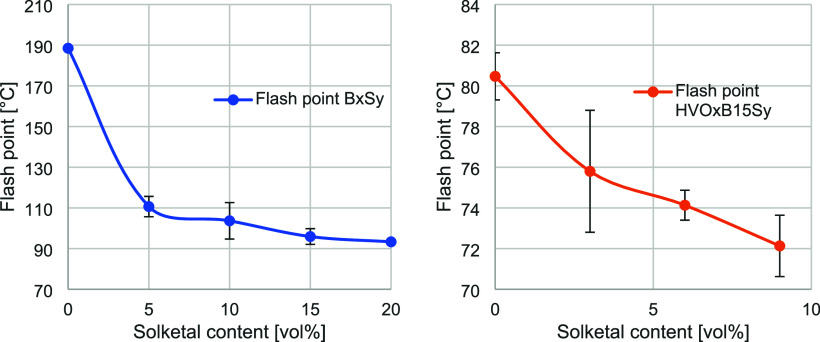
Trends of the flash point with increasing solketal content for the binary system BxSy (left, blue) and the ternary system HVOxB15Sy (right, orange) (*n* = 3).

The observation that the addition of a component with a lower flash point reduces the overall flash point of a blend is well established. This behavior has also been reported for biodiesel containing methanol.^52^ In the present study, a pronounced reduction was observed in the binary mixtures. The addition of 5 vol % solketal resulted in a 41% decrease in flash point. This was followed by a gradual leveling off, although further minor decreases were still evident (from approximately 6–7% down to 2.3%). In contrast, the ternary mixtures exhibited a more linear trend, with reductions ranging from 2 to 6%, except for a fluctuation observed at 3 vol % solketal. Due to the lower solketal content, an error caused by miscibility should be excluded. The other error ranges of the blends in Figure 6 showed a smaller deviation, suggesting that the plots were reasonable. The higher linearity in the ternary system can be attributed to the presence of HVO, which already induced a lower initial flash point even without solketal addition. The initial flash point values prior to solketal blending differed by 54.3%. Overall, these findings indicate that a more detailed physical investigation of the evaporation behavior of solketal is warranted.

### Influence of Solketal on the Vapor Pressure of Fuel Components

#### Vapor Pressure of Pure Solketal and Mixtures with Similar Fuel Components

First, the vapor pressure curve of pure solketal was recorded. For comparison, vapor pressure curves of methanol and ethanol were also analyzed. Both alcohols were selected due to their similar fuel-related properties to solketal, resulting from comparable oxygen-to-carbon ratios and chemical functionalities. However, when examining the vapor pressure, it becomes evident that solketal differs significantly from methanol and ethanol. The corresponding curves are presented in Figure 7.

**Figure 7:**
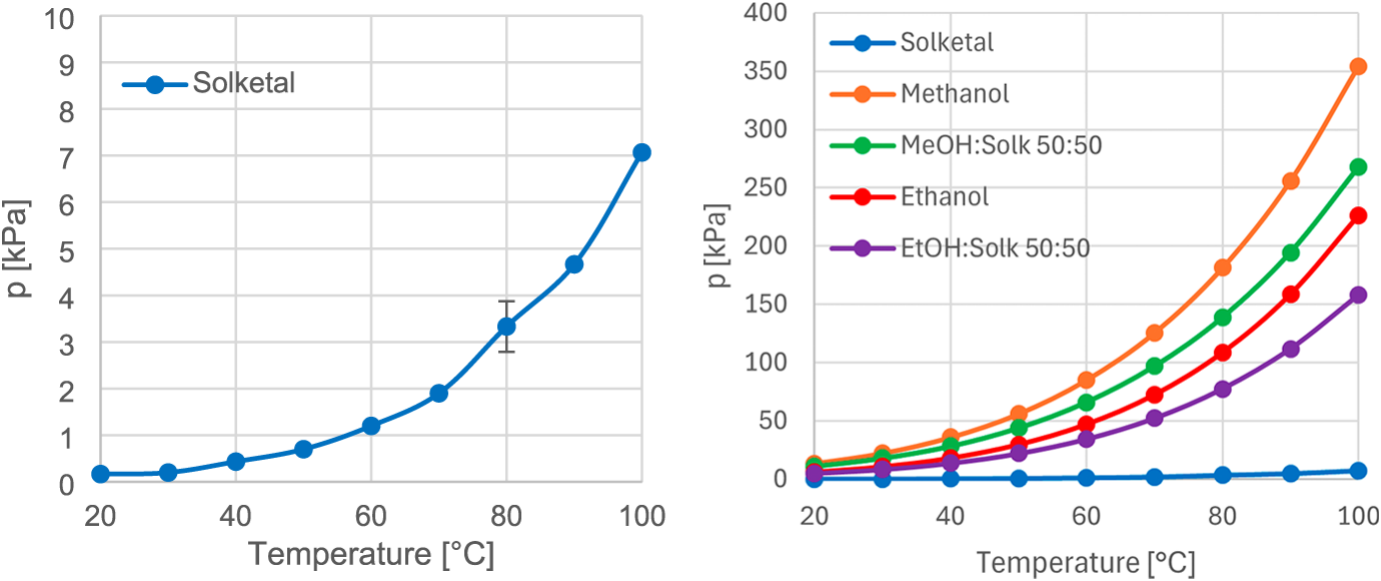
Vapor pressure curves of pure solketal (Solk, left and right, blue), methanol (MeOH, right, orange), MeOH–solketal mixture (50:50, right, green), ethanol (EtOH, right, red), and EtOH–solketal mixture (50:50, right, violet). The left graph presents a magnified view to more clearly illustrate the exponential increase. The right graph compares the respective trends to highlight the differences in their orders of magnitude (*n* = 3).

It was evident that solketal exhibited a significantly lower vapor pressure curve compared to the reference alcohols. Subsequently, the vapor pressures at 100 °C were compared, as this temperature provided the most suitable basis for comparison due to the exponential increase in vapor pressure with temperature. The vapor pressure of solketal was lower than that of methanol by a factor of 50.1 and lower than that of ethanol by a factor of 32. One approach to increasing vapor pressure is the addition of a component with a higher inherent vapor pressure. For this purpose, 50:50 mixtures of solketal with methanol and ethanol were prepared. In the solketal–methanol system, solketal exhibited an increase in vapor pressure by a factor of 37.9, while methanol showed a decrease of 0.76. In the solketal–ethanol system, solketal exhibited an increase by a factor of 32, accompanied by a decrease of 0.7 for pure ethanol. These results demonstrate that solketal exerts a comparable influence in both systems. Table 5 presents a comparison between the theoretically calculated total partial pressures (*p*_theor_) and the experimentally determined pressures (*p*_exp_) at 30 °C. This temperature was selected to minimize thermal effects and to allow assessment of whether the mixtures exhibit ideal or nonideal behavior. The deviation is described by the following equation:

$$p_{\mathrm{deviation}}=\frac{p_{\exp}-p_{\mathrm{theor}}}{p_{\mathrm{theor}}}$$

Both mixtures exhibited a positive deviation, indicating weaker intermolecular interactions between the respective components. The closer the activity coefficient (γ) approaches unity, the more ideally a system behaves. Accordingly, the methanol–solketal mixture displayed nearly ideal behavior, whereas the solketal–ethanol system showed a higher degree of nonideality. This observation is consistent with the molecular structure of ethanol, whose longer carbon chain reduces the effectiveness of hydrogen bonding through steric effects. Furthermore, there is a general tendency for the deviation from ideal behavior to increase with increasing alkyl chain length.

The measured RVP of solketal was 0 kPa (*n* = 3), indicating poor cold-starting capability. In the binary mixtures, the addition of methanol resulted in an RVP of 24.5 kPa (*n* = 3; pure methanol: 31.8 kPa), while the addition of ethanol yielded an RVP of 11.1 kPa (*n* = 3; pure ethanol: 15.6 kPa). Overall, the RVP values for all alcohol-containing mixtures remained below the typical range of 45–90 kPa. Consequently, blending is required to enhance the vapor pressure of solketal. The results suggest that methanol and ethanol act as covolatile components, increasing the fraction of solketal present in the vapor phase.

#### Vapor Pressure of Solketal/Biodiesel System

The investigations with methanol and ethanol demonstrated that binary systems could lead to an increase in the vapor pressure of solketal. Compared to other established fuels, biodiesel exhibits a low vapor pressure, a relatively high boiling point, and therefore an increased tendency to dilute engine oil.^53^ A comparison of the pure components revealed that solketal did not show a significant increase in vapor pressure relative to biodiesel (factor 2.02 at 100 °C). However, it was reasonable to assume that the formation of a binary mixture could result in an overall increase in vapor pressure. The results obtained with methanol and ethanol indicated that nonideal behavior increases with higher nonpolar content, i.e., with elongation of the carbon chain, due to weaker polar interactions. This suggests that biodiesel can be utilized to selectively enhance the volatility of solketal and, conversely, that solketal may increase the volatility of biodiesel.

The activity coefficient was estimated qualitatively, as an exact evaluation would have required experimental determination of the partial pressures of all individual biodiesel components. Nevertheless, qualitative trends could be identified. In this model, biodiesel was treated as a homogeneous substance with an assigned single partial pressure contributing to the total vapor pressure of the system. For the rapeseed methyl ester (RME) used, a mean molar mass of 323.4 g mol^–1^ was assumed based on the work of Peterson et al.^54^ In order to examine this influence, solketal was added to biodiesel in 5 vol % increments up to 20 vol %. The corresponding vapor pressure curves are shown in Figure 8.

**Figure 8:**
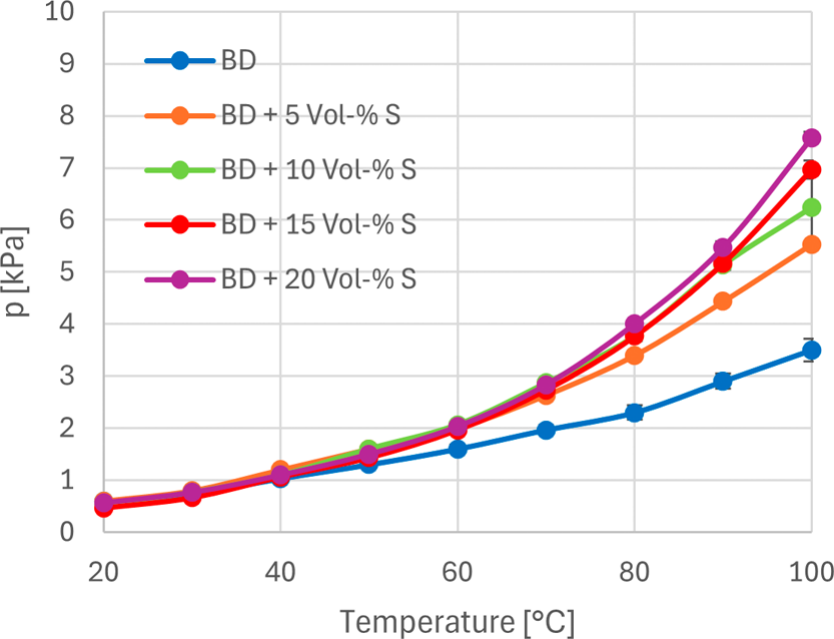
Vapor pressure curves of biodiesel (blue) and biodiesel–solketal blends with 5 vol % (orange), 10 vol % (green), 15 vol % (red), and 20 vol % (violet) solketal content (*n* = 3).

It was evident that the addition of solketal resulted in an increase in the vapor pressure of biodiesel. Up to 40 °C, the vapor pressure curves showed negligible differences; however, between 50 and 100 °C, the curves began to diverge progressively. In order to generate a basis for comparison, the vapor pressures at 100 °C were examined. With increasing solketal content, the vapor pressure increased continuously. The increase in each solketal blend was compared with pure biodiesel. The increase was determined by dividing the vapor pressure of the solketal blend by the vapor pressure of pure biodiesel. The most pronounced rise occurred in the transition from pure biodiesel to the initial solketal blend (factor 1.6). Each subsequent increase in solketal concentration produced an additional rise in the factor of approximately 0.2. Notably, the blend containing 15 vol % solketal nearly reached the vapor pressure of pure solketal (6.97 compared to 7.06), and at 20 vol % solketal, the blend even exhibited a higher vapor pressure than pure solketal itself (factor 1.07). To evaluate whether the binary mixtures behaved ideally or nonideally, the activity coefficient (γ) was calculated for the respective blends, as shown in Table 6.

**Table 6:** Determination of the Deviation and the Activity Coefficient (γ) of the Binary Biodiesel–Solketal System

Solketal content [vol %]	*p*_theor_ at 100 °C [kPa]	*p*_exp_ at 100 °C [kPa]	*p*_deviation_ [%]	Activity coefficient γ
5	3.99	5.53	39	1.39
10	4.38	6.23	42	1.42
15	4.72	6.97	48	1.48
20	5.01	7.57	51	1.51

Although the system was idealized in terms of partial pressures and molecular mass, the magnitude of the activity coefficient (γ) indicates that the mixtures exhibit nonideal behavior. Surface tension measurements of the binary solketal–biodiesel mixtures suggest that changes in surface tension are unlikely to be a decisive factor in this context. Based on the results and observations from the determination of γ, it can be inferred that the longer carbon chains of the biodiesel components reduce the strength of intermolecular interactions. Despite the limited suitability of biodiesel–solketal blends for application in gasoline engines, the RVP was measured. For all samples tested (*n* = 3), an RVP of 0 kPa was obtained, confirming their unsuitability for such use.

#### Vapor Pressure of Solketal/Gasoline Fuel Blends

After examining the influence of solketal on the vapor pressure of a renewable fuel component such as biodiesel, its effect on gasoline fuels was subsequently analyzed. Apart from the vapor pressure, the increased boiling range of solketal also implies more challenging boiling behavior. Vapor pressure, particularly the Reid vapor pressure (RVP), represents a key parameter defined in the EN 228 fuel standard (see Supporting Information). Owing to its favorable properties as a potential gasoline fuel component, the impact of solketal on the vapor pressure of gasoline was investigated. Figure 9 presents the corresponding vapor pressure curves for pure gasoline and for blends containing 10 and 20 vol % solketal, while Table 7 summarizes the factors describing the reduction in vapor pressure induced by solketal addition.

**Figure 9:**
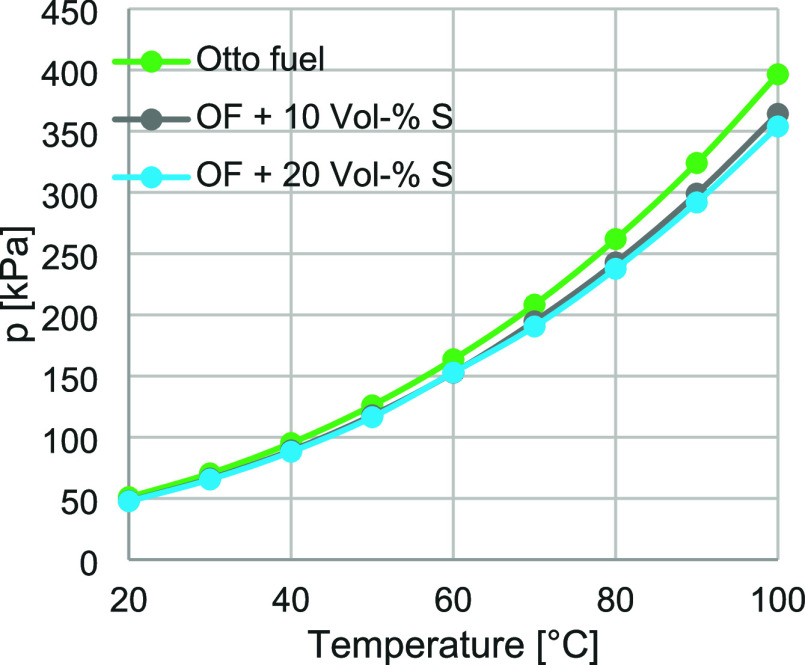
Vapor pressure curve for fossil gasoline (OK, light green), a blend with 10 vol % solketal (gray), and a blend with 20 vol % solketal (light blue) (*n* = 4).

**Table 7:** Calculation of the Decrease in Vapor Pressure at 100 °C Resulting from Blending Solketal into Fossil Gasoline; the RVP of the Blends Is Also Reported

Solketal content [vol %]	Vapor pressure at 100 °C [kPa]	Decrease	RVP [kPa]
0	396.5	/	88.4 ± 0.45
10	364.2	0.08	83.13 ± 0.46
20	353.9	0.11	82.33 ± 0.61

The reduction in vapor pressure was 0.08 for the blend containing 10 vol % solketal and 0.11 for the blend containing 20 vol %. Since fossil gasoline fuels exhibit substantially more heterogeneous behavior due to their complex hydrocarbon composition and the presence of additional chemical functionalities such as olefins and aromatics, a simplified description of vapor pressure and molar mass was not applied. The relatively small decreases also indicate the formation of a nonideal mixture. Similar to the observations made with methanol, ethanol, and biodiesel, it is conceivable that the hydrocarbons in gasoline interact less effectively with solketal, leading to a higher proportion of solketal in the vapor phase. The unblended fossil gasoline fuel exhibited an RVP of 88.4 kPa, indicative of good cold-starting behavior. Since solketal caused only a slight reduction in RVP (by a factor of 0.06 at 10 vol % and 0.07 at 20 vol %), the cold-start behavior can be considered drop-in capable for gasoline engine applications. Furthermore, the low intrinsic vapor pressure of solketal in the blend appears to be compensated by the higher volatility of the gasoline hydrocarbons, resulting in a sufficiently high overall vapor pressure. Regarding the distillation range and other gasoline parameters, studies by Alptekin and Canakci have shown that 9 vol % of solketal complied with the EN 228 standard.^26^

## Conclusion and Outlook

The investigation of the physical and chemical fuel properties enabled a detailed characterization of the influence of solketal on blend stability, key parameters defined in the EN 590, and vapor pressure. The focus was placed on examining miscibility gaps and the associated effects of polarity. The main influences of solketal on the EN 590 parameters were identified primarily in density, ignition behavior, and evaporation characteristics. As the increasing use of renewable fuel components such as HVO reduces fuel density, higher-density components such as solketal are desirable to counteract this effect. Furthermore, the conversion of the biodiesel byproduct glycerol into solketal, followed by its use as a fuel additive, represents an effective approach to increase the value of this byproduct.

The ternary blend systems investigated for miscibility gaps demonstrated that the hydrocarbon matrix used is decisive for determining the amount of solketal that can be blended. The comparison between DF and HVO revealed that the aromatic content plays a critical role. With an aromatic content of 18.84 wt %, no binary blends between DF and solketal were feasible, whereas an aromatic content of 24.4 wt % was sufficient to achieve miscibility. Biodiesel acted as a solubilizing agent. The absence of aromatics in HVO enabled a clearer assessment of the interactions between diesel hydrocarbons and solketal. Stable blends were observed at 7 vol % biodiesel with 1 vol % solketal and at 24 vol % biodiesel with up to 7 vol % solketal. The more challenging blending behavior can be attributed to the increase in polarity, reflected by a rise in permittivity of 15.7% in the BxSy system and 9.8% in the HVOxB15Sy system. Simultaneously, the interfacial tension decreased by 43.6% and 68.3%, respectively.

Moreover, the influence of solketal on the blends was also evident in changes to density, flash point, and ignition characteristics. While nearly linear trends were observed for density (increase) and CN (decrease), the flash point and CI exhibited nonlinear decreases with increasing solketal content. The relationship between density, ignition, and evaporation behavior subsequently directed attention toward the vapor pressure characteristics. Vapor pressure curves were analyzed for pure solketal, 50:50 mixtures with methanol and ethanol, as well as for binary systems with biodiesel and fossil gasoline at varying concentrations. The activity coefficients calculated according to Raoult’s law confirmed the nonideal mixing behavior of the systems, with the methanol–solketal mixture showing behavior closest to ideality. It was also observed that the degree of nonideal behavior increased with the length of the carbon chain. A complementary investigation of the RVP measurements demonstrated that solketal alone exhibits poor cold-start behavior in gasoline engines. However, in binary blends with fossil gasoline (10 and 20 vol %), the measured RVP values were within a range consistent with drop-in capability.

Following the investigation of solketal’s drop-in capability in this work, further research steps are required. In particular, the influence of aromatic compounds should be examined systematically. Moreover, the maximum allowable solketal blending ratio that ensures compliance with fuel standards should be defined. The observed reduction in interfacial tension also highlights the importance of investigating water separation behavior in greater detail. It is also necessary to conduct further research into the water absorption capacity and hygroscopicity of fuels containing solketal. In addition, the material compatibility and injector durability must be further addressed. Finally, interactions between solketal-containing fuels and engine oils should be evaluated for both chemical compatibility and dilution effects.

## Supplementary Material



## Notes

Ternary blend diagrams (0–100 vol %), list of experiments, and the fuel analysis (Tables S1–S12 and Figures S1–S3) (PDF)

## References

[1] O’Connell N. (2025). Potential of DMC and PODE as Fuel Additives for Industrial Diesel
Engines. Fuels.

[2] Perkins. Renewable fuels for use in diesel engines; 2026, https://www.perkins.com/en_GB/company/sustainability/renewable-and-low-carbon-intensity-fuels-for-use-in-diesel-engines.html.

[3] Götz, K. ; Fey, B. ; Singer, A. ; Krahl, J. Exhaust Gas Emissions and Engine Oil Interactions from a New Biobased Fuel Named Diesel R33 2016-01-2256; 2016, SAE International

[4] Lapuerta M. (2011). Key properties and blending strategies of hydrotreated vegetable
oil as biofuel for diesel engines. Fuel Process.
Technol..

[5] Zeman P. (2019). Hydrotreated vegetable oil as a fuel from waste materials. Catalysts.

[6] Bjørgen K. O. P., Emberson D. R., Løvås T. (2020). Combustion and soot characteristics
of hydrotreated vegetable oil compression-ignited spray flames. Fuel.

[7] Zhai C. (2024). Experimental Study on
the Spray Characteristics of Diesel and Hydrotreated
Vegetable Oil (HVO) Fuels under Different Injection Pressures. Processes.

[8] Wójcik J. K., Główka M., Boberski P., Postawa K., Jaroszewska K. (2025). The importance
of hydroisomerization catalysts in development of sustainable aviation
fuels: Current state of the art and challenges. J. Ind. Eng. Chem..

[9] Buelens L. C. (2016). Super-dry reforming
of methane intensifies CO2 utilization via Le
Chatelier’s principle. Science.

[10] Wu C., Huang Q., Xu Z., Sipra A. T., Gao N., de Souza Vandenberghe L. P., Vieira S., Soccol C. R., Zhao R. (2024). A comprehensive
review of carbon capture science and
technologies. Carbon Capture Sci. Technol..

[11] Neoh K. G., Howard J. B., Sarofim A. F. (1985). Effect of oxidation
on the physical
structure of soot. Symp. (Int.) Combust..

[12] Omari A. (2019). Potential of long-chain
oxymethylene ether and oxymethylene ether-diesel
blends for ultra-low emission engines. Appl.
Energy.

[13] Lucas S. P. (2022). Fuel properties of oxymethylene ethers with terminating groups from
methyl to butyl. Energy Fuels.

[14] Zhang, X. ; Li, L. ; Wu, Z. ; Hu, Z. Material Compatibilities of Biodiesels with Elastomers, Metals and Plastics in a Diesel Engine 2009-01-2799; SAE International, 2009.

[15] Goosen, R. ; Vora, K. ; Vona, C. Establishment of the guidelines for the development of biodiesel standards in the APEC region; APEC Biodiesel Standard EWG, 2007, 74, 53–55.

[16] Arouni H. (2018). Limitations of monoolein in simulating water-in-fuel
characteristics
of EN590 diesel containing biodiesel in water separation testing. SAE Int. J. Fuels Lubr..

[17] Ra Y., Reitz R. D., Mcfarlane J., Daw C. S. (2009). Effects of fuel
physical properties on diesel engine combustion using diesel and bio-diesel
fuels. SAE Int. J. Fuels Lubr..

[18] Liu Y., Zhong B., Lawal A. (2022). Recovery and
utilization of crude
glycerol, a biodiesel byproduct. RSC Adv..

[19] Oleoline, H. Glycerine market report; Oleoline, 2025.

[20] Steinmetz S. A., Herrington J. S., Winterrowd C. K., Roberts W. L., Wendt J. O. L., Linak W. P. (2013). Crude
glycerol combustion: Particulate,
acrolein, and other volatile organic emissions. Proc. Combust. Inst..

[21] Stelmachowski M. (2011). Utilization
of glycerol, a by-product of the transestrification process of vegetable
oils: a review. Ecol. Chem. Eng..

[22] Coronado C. R. (2014). Ecological efficiency
in glycerol combustion. Appl. Therm. Eng..

[23] Jaecker-Voirol A., Durand I., Hillion G., Delfort B., Montagne X. (2008). Glycerin for new biodiesel formulation. Oil
Gas Sci. Technol..

[24] Nanda M. R. (2016). Catalytic conversion of glycerol for sustainable production of solketal
as a fuel additive: A review. Renewable Sustainable
Energy Rev..

[25] Mota C. J. (2010). Glycerin derivatives as fuel additives: The addition of glycerol/acetone
ketal (solketal) in gasolines. Energy Fuels.

[26] Alptekin E., Canakci M. (2017). Performance and emission
characteristics of solketal-gasoline
fuel blend in a vehicle with spark ignition engine. Appl. Therm. Eng..

[27] Vichare M. S., Chakraborty M., Jana A. K. (2023). Engine performance study for solketal-gasoline
fuel blend in a four-stroke SI engine. Clean
Technol. Environ. Policy.

[28] Vichare M. S., Chakraborty M., Jana A. K. (2025). Solketal-diesel nanoemulsion as fuel
for diesel engine: a study on the emulsion stability, engine performance,
and emission characteristics. J. Dispersion
Sci. Technol..

[29] Lin C.-Y., Tsai S.-M. (2020). Emission characteristics
of a diesel engine fueled
with nanoemulsions of continuous diesel dispersed with solketal droplets. J. Environ. Sci. Health, Part A.

[30] Türck J., Riess S., Strauß L., Schmitt F., Türck R., Ruck W., Wensing M., Krahl J. (2025). Investigation of the
spray formation of solketal under diesel engine conditions and the
influence on Diesel R33. Fuel Process. Technol..

[31] Tuerck J., Singer A., Lichtinger A., Almaddad M., Türck R., Jakob M., Garbe T., Ruck W., Krahl J. (2022). Solketal as
a renewable fuel component in ternary blends with biodiesel and diesel
fuel or HVO and the impact on physical and chemical properties. Fuel.

[32] Türck J., Schmitt F., Anthofer L., Türck R., Ruck W., Krahl J. (2023). Extension of Biodiesel
aging mechanism–the
role and influence of Methyl Oleate and the contribution of Alcohols
through the use of Solketal. ChemSusChem.

[33] Türck, J. Wechselwirkung und Einfluss von Solketal auf die Alterung von Fettsäuremethylestern. Kraftstoffe für die Mobilität von morgen: 4. Tagung der Fuels Joint Research Group am 10. und 11.Juni 2021 in Dresden-Radebeul, vol. 30; 2021.

[34] Kerkel F., Brock D., Touraud D., Kunz W. (2021). Stabilisation of biofuels
with hydrophilic, natural antioxidants solubilised by glycerol derivatives. Fuel.

[35] Munack, A. ; Schmidt, L. ; Schröder, O. ; Schaper, K. ; Pabst, C. ; Krahl, J. Alcohols as a means to inhibit the formation of precipitates in blends of biodiesel and fossil diesel fuel. Agric. Eng. Int. 2015.

[36] Saikia K., Rajkumari K., Moyon N. S., Basumatary S., Halder G., Rashid U., Rokhum S. L. (2022). Sulphonated biomass-based
catalyst for solketal synthesis by acetalization of glycerol–A
byproduct of biodiesel production. Fuel Process.
Technol..

[37] Dmitriev G. S., Terekhov A. V., Zanaveskin L. N., Maksimov A. L., Khadzhiev S. N. (2018). Kinetics
of the Formation of Solketal in the Presence of Sulfuric Acid. Kinet. Catal..

[38] Esteban J., Ladero M., García-Ochoa F. (2015). Kinetic modelling
of the solventless
synthesis of solketal with a sulphonic ion exchange resin. Chem. Eng. J..

[39] Royon D., Locatelli S., Gonzo E. E. (2011). Ketalization of
glycerol to solketal
in supercritical acetone. J. Supercrit. Fluids.

[40] Fernando S., Hanna M. (2004). Development of a novel biofuel blend using ethanol– biodiesel–
diesel microemulsions: EB-diesel. Energy Fuels.

[41] Assaf K. I., Nau W. M. (2023). Dispersion interactions
in condensed phases and inside
molecular containers. Acc. Chem. Res..

[42] Hwang J. (2015). How important are dispersion interactions to the strength of aromatic
stacking interactions in solution?. Chem. Sci..

[43] Karimi M., Parsafar G., Samouei H. (2024). Polarizing
Perspectives: Ion-and
Dipole-Induced Dipole Interactions Dictate Bulk Nanobubble Stability. J. Phys. Chem. B.

[44] Sherrill C. D. (2013). Energy
component analysis of π interactions. Acc. Chem. Res..

[45] Ghosh S., Ray A., Pramanik N. (2020). Self-assembly
of surfactants: An overview on general
aspects of amphiphiles. Biophys. Chem..

[46] Bowden, J. N. ; Johnston, A. A. ; Russell, J. A. Octane-cetane relationship; Defense Technical Information Center, 1974.

[47] Kircher M. (2023). Investigation of engine combustion and auto-ignition
of a multicomponent
surrogate fuel with NTC behavior under knocking conditions. Flow, Turbul. Combust..

[48] Vasileiadis V. (2025). Mathematical Correlations
for Volumetric (Density and Specific Gravity)
Properties of Diesel/Biodiesel Blends. Appl.
Sci..

[49] Glaconchemie GmbH. Glycasol, 2026, https://glaconchemie.de/produkte/glycerin-derivate/glyca-sol.

[50] Türck J. (2023). Oxidation Kinetics of
Neat Methyl Oleate and as a Blend with Solketal. Energies.

[51] El-Seesy A.
I. (2021). Impacts
of octanol and decanol addition on the solubility of methanol/hydrous
methanol/diesel/biodiesel/Jet A-1 fuel ternary mixtures. RSC Adv..

[52] Wang Z. (2020). Characterization of
biomethanol–biodiesel–diesel blends
as alternative fuel for marine applications. J. Mar. Sci. Eng..

[53] Reddy V. M., Biswas P., Garg P., Kumar S. (2014). Combustion
characteristics of biodiesel fuel in high recirculation conditions. Fuel Process. Technol..

[54] Peterson C. L., Reece D. L., Hammond B. L., Thompson J., Beck S. M. (1997). Processing,
characterization, and performance of eight fuels from lipids. Appl. Eng. Agric.

