# Supplementary material for: Drop-In Capability of Solketal in Diesel and Gasoline Fuels Containing Biodiesel, HVO, Fossil Diesel, and Gasoline Based on Standard Physical and Chemical Fuel Properties
Source: ACS Omega. 2026 Apr 16;11(16):24063–74. doi: 10.1021/acsomega.5c12056 (PMC13130121; doi:10.1021/acsomega.5c12056)
Supplement: Supplementary file 1 [file ao5c12056_si_001.pdf]

Supporting Information

Drop-in capability of solketal in diesel and gasoline fuels containing biodiesel, HVO, fossil diesel and gasoline based on standard physical and chemical fuel properties.

Julian Türck[a,b]*, Fabian Schmitt[b], Sumit Agarwal[c], Jens Utecht[d], Ralf Türck[b,e], Wolfgang Ruck[a] and Jürgen Krahl[e,f]

[a] *Leuphana University Lüneburg, School of Sustainability, Universitätsallee 1, 21335 Lüneburg, Germany*

[b] *Tecosol GmbH, Jahnstraße 2, 97199 Ochsenfurt, Germany*

[c] *Department of Physical Chemistry, Physikalisch-Technische Bundesanstalt, Bundesallee 100, Braunschweig, 38116, Germany*

[d] *L.M.U. Business Consulting GmbH, Im Baumgarten 22, 67283 Obrigheim, Germany*

[e] *Fuels Joint Research Group, www.fuels-jrg.de, Germany*

[f] *OWL University of Applied Sciences and Arts, Campusallee 12, 32657 Lemgo, Germany*

*Email: Julian.tuerck@stud.leuphana.de

Table of contents

1 Ternary blend diagrams (0-100 vol%) S2

2 List of experiments S3

3 Fuel analysis

3.1 Miscibility gap S4

3.2 EN590 S7

3.3 Solketal influence S10

3.4 Vapor pressure S14

Abbreviations

B0	Fossil diesel fuel
CFPP	Cold filter plugging point
DF	Diesel fuel
FAME	Fatty acid methyl ester
HFRR	High frequency reciprocating rig
PUFA	Polyunsaturated fatty acid
HVO	Hydrotreated vegetable oil

1 Ternary blend diagrams (0-100 vol%)

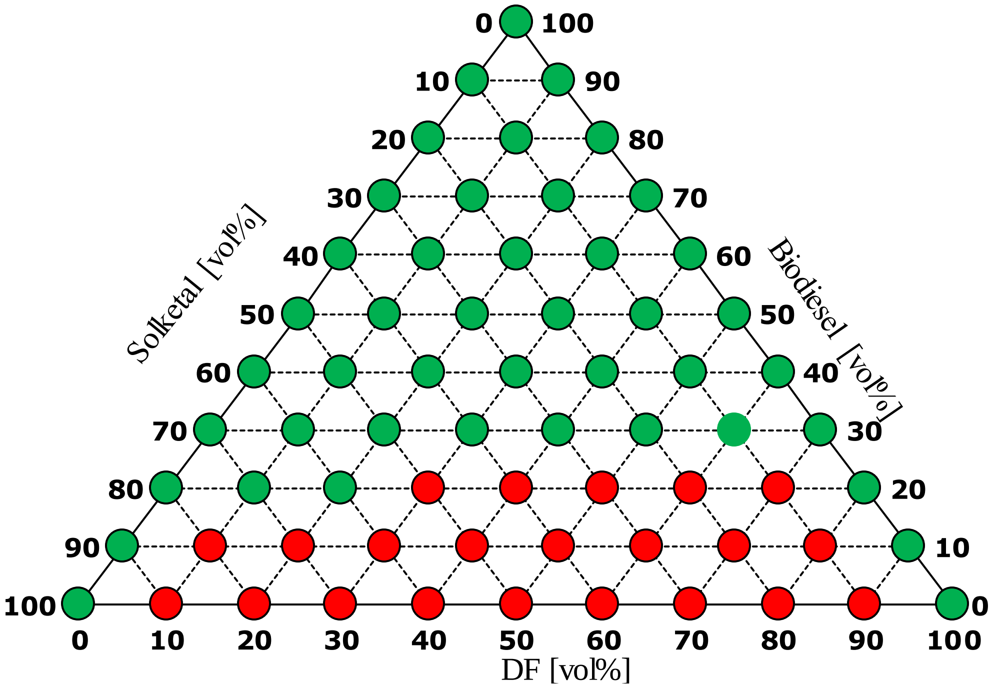

Figure S1*:* *Ternary mixture diagram of DF, biodiesel, and solketal. The mixing range was between 0 and 100 vol% (in 10 vol% increments). The green markers represent stable single-phase blends, while the red markers indicate unstable two-phase blends.*

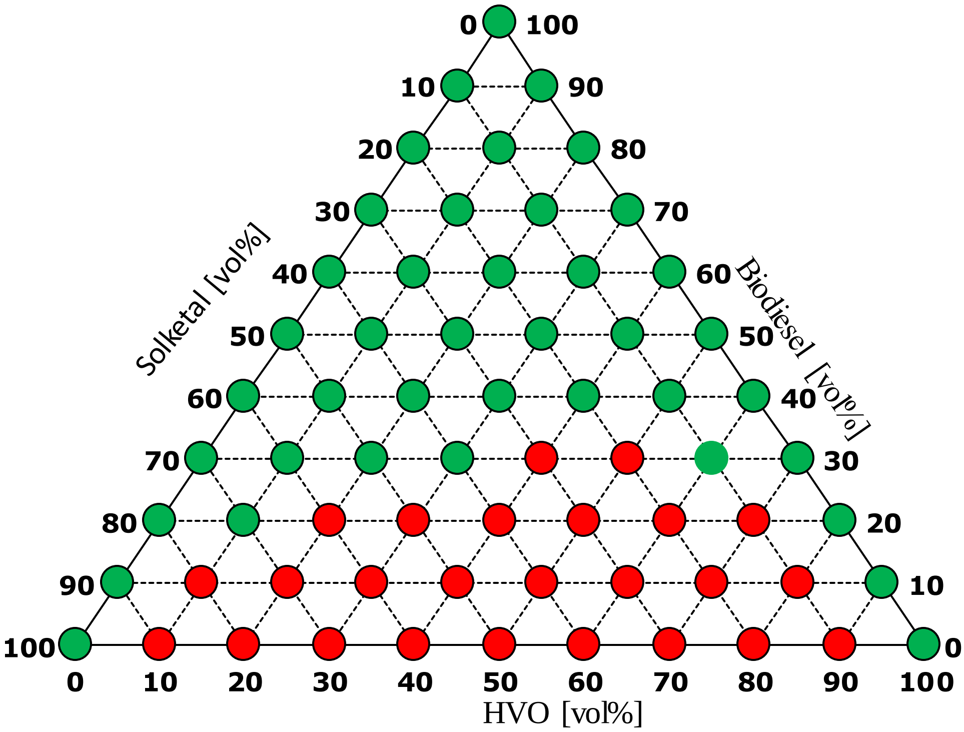

Figure S2*:* *Ternary mixture diagram of HVO, biodiesel, and solketal. The mixing range was between 0 and 100 vol% (in 10 vol% increments). The green markers represent stable single-phase blends, while the red markers indicate unstable two-phase blends.*

2 List of experiments

Table S1*: Overview of the EN590 parameters together with the corresponding analytical methods. Furthermore, it is specified whether each measurement was performed internally or externally.*

Parameter	Method	Internally	Externally
Cetane number	DIN EN 17155 : 2018		■
Cetane index	DIN EN ISO 4264 : 2018		■
Density (15 °C)	DIN EN ISO 12185 : 1997	■	
Polycyclic aromatic hydrocarbons	DIN EN ISO 12916 :2019		■
Sulfur content	DIN EN ISO 20846 : 2019	■	
Flash point	DIN EN ISO 2719 : 2016	■	
Coke residue (10% D.)	DIN EN ISO 10370 : 2015		■
Ash content (775 °C)	EN ISO 6245 : 2003		■
Water content	DIN EN ISO 12937 : 2002	■	
Total contamination	DIN EN 12662 : 1998	■	
Corrosion effect on copper	DIN EN ISO 12205 : 1996		■
Oxidation stability	DIN EN 15751 : 2014	■	
HFRR (lubricity at 60°C)	DIN EN ISO 12156 :2019		■
Kinetic viscosity (40 °C)	DIN EN ISO 16896	■	
Volume at 25°C			■
Volume at 350 °C	DIN EN ISO 3924 : 2019		■
95% point			■
CFPP	DIN EN 116 :2015	■	
Manganese (Mn)	DIN EN 16576 : 2015		■

3 Fuel analysis

3.1 Miscibility gap

Biodiesel

Table S2: *Fuel analysis of the biodiesel (rapeseed methyl ester) supplied by ASG-Analytik-Service GmbH (Germany) for the miscibility gap investigation.*

Parameter	Method	Result	Unit
Density at 15 °C	DIN EN ISO 12185 :1997	881.3	kg/m³
Viscosity at 40 °C	DIN EN ISO 3104 :2021	4.256	mm²/s
Flash point	DIN EN ISO 2719 :2021	186.5	°C
CFPP	DIN EN 116 :2018	-9	°C
Sulphur content	DIN EN ISO 20884 :2022	<5(1.3)	mg/kg
Cetane number	DIN EN 17155 :2018	51.7	-
Ash content (sulphate ash)	ISO 3987 :2010	<0.01	% (m/m)
Water content (Karl Fischer)	DIN EN ISO 12937 :2002	40	mg/kg
Total pollution	DIN EN 12662 :1998	<1	mg/kg
Corrosion effect on copper	DIN EN ISO 2160 :1999	1	Korr. Grad
Oxidation stability 110° C	DIN EN 14112 :2021	1.8 │1.9	h
Acid value	DIN EN 14104 :2003	0.24	mg KOH /g
Iodine value	DIN EN 16300: 2012	110.2	g Iod /100g
PUFA	DIN EN 15779 :2013	<0.60	% (m/m)
Methanol content	DIN EN 14110 :2011	<0.01	% (m/m)
Free glycerine content	DIN EN 14105	0.008	% (m/m)
Monoglyceride content		0.11	% (m/m)
Diglyceride content		<0.01	% (m/m)
Triglyceride content		<0.01	% (m/m)
Total glycerine content	DIN EN 14105	0.037	% (m/m)
Alkali metal content (Na+K)	DIN EN 14538: 2006	<1	mg/kg
Alkaline earth metal content (Ca+Mg.)		<1	mg/kg
Phosphorus content	DIN EN 14107 :2003	<4 (<0.5)	mg/kg
Cloudpoint	DIN EN 23015: 1994	-8	°C

HVO

***Table S3**: Fuel analysis of the HVO supplied by ASG-Analytik-Service GmbH (Germany) for the miscibility gap investigation.*

Parameter	Method	Result	Unit
Cetane number	DIN EN 17155 :2018	74.8	-
Density at 15°C	DIN EN ISO 12185 :1997	780.5	kg/m³
Flash point	DIN EN ISO 2719 :2021	74.5	°C
Kin. Viscosity a140°C	DIN EN ISO 3104 :2021	2.973	mm²/s
Volume at 250 °C	DIN EN ISO 3924 :2019	4.5	% (V/V)
Volume at 350 °C		-	% (V/V)
95%-Point		296.0	°C
HFRR (Avg.)	DIN EN ISO 12156-1 :2019	432	µm
FAME content	DIN EN 14078 :2014	<0.01	% (V/V)
Manganese	DIN EN 16576 :2015	<0.50	mg/l
Total aromatic content	DIN EN 12916 Verf. B	<0.1	% (m/m)
Sulfur	DIN EN ISO 20884 :2022	<5(<1)	mg/kg
Coke residue	DIN EN ISO 10370 :2015	<0.1	% (m/m)
Ash content	DIN EN ISO 6245 :2003	<0.001	% (m/m)
Water content	DIN EN ISO 12937 :2002	<30	mg/kg
Total pollution	DIN EN 12662 :2014	<12	mg/kg
Corrosion - Copper	DIN EN ISO 2160 :1999	1	Korr. Grad
Filterable aging residue	DIN EN ISO 12205 :1996	<1	g/m³
Non filterable aging residue		<1	g/m³
Oxidation stability		<1	g/m³
Oxidation stability	DIN EN 15751 :2014	-	h
CFPP	DIN EN 116 :2018	-24	°C
Heating value	DIN 51900-2 :2003 mod.	43.6	MJ/kg
Carbon	DIN 51732 :2014	84.84	% (m/m)
Hydrogen		15.41	% (m/m)
Nitrogen		<0.5	% (m/m)

B0

Table S4: *Fuel analysis of the B0 supplied by ASG-Analytik-Service GmbH (Germany) for the miscibility gap investigation.*

Parameter	Method	Result	Unit
Cetane number	DIN EN 17155 :2018	54	-
Cetane index	DIN EN ISO 4264 :2018	53.6	-
Density at 15 C	DIN EN ISO 12185 :1997	832.7	kg/m³
Aromatics, Poly (2+3)	DIN EN 12916 :2022 Verf. A	1.6	% (m/m)
Sulfur	DIN EN ISO 20884 :2022	8.3	mg/kg
Flash point	DIN EN ISO 2719 :2021	66	°C
Coke residue	DIN EN ISO 10370 :2015	<0.1	% (m/m)
Ash content	DIN EN ISO 6245 :2003	0.002	% (m/m)
Water content	DIN EN ISO 12937 :2002	<30	mg/kg
Total pollution	DIN EN 12662 :2014	<12(1)	mg/kg
Corrosion - Copper	DIN EN ISO 2160 :1999	1	Korr. Grad
FAME content	DIN EN 14078 :2014	<0.1	% (V/V)
Oxidation stabillity	DIN EN ISO 12205 :1996	4	g/m³
filterable aging residue		<1	g/m³
non filterable aging residue		4	g/m³
Oxidation stabillity	DIN EN 15751 :2014	-	h
HFRR	DIN EN ISO 12156-1 :2019	380	µm
Kin. Viscosity at 40 °C	DIN EN ISO 3104 :2021	2.815	mm²/s
Volumen at 250 °C	DIN EN ISO 3924 :2019	37	% (V/V)
Volume at 350 °C		91.7	% (V/V)
95 %-Point		360.8	°C
CFPP	DIN EN 116 :2018	-24	°C
Manganese	DIN EN 16576 :2015	<0.5	mg/l

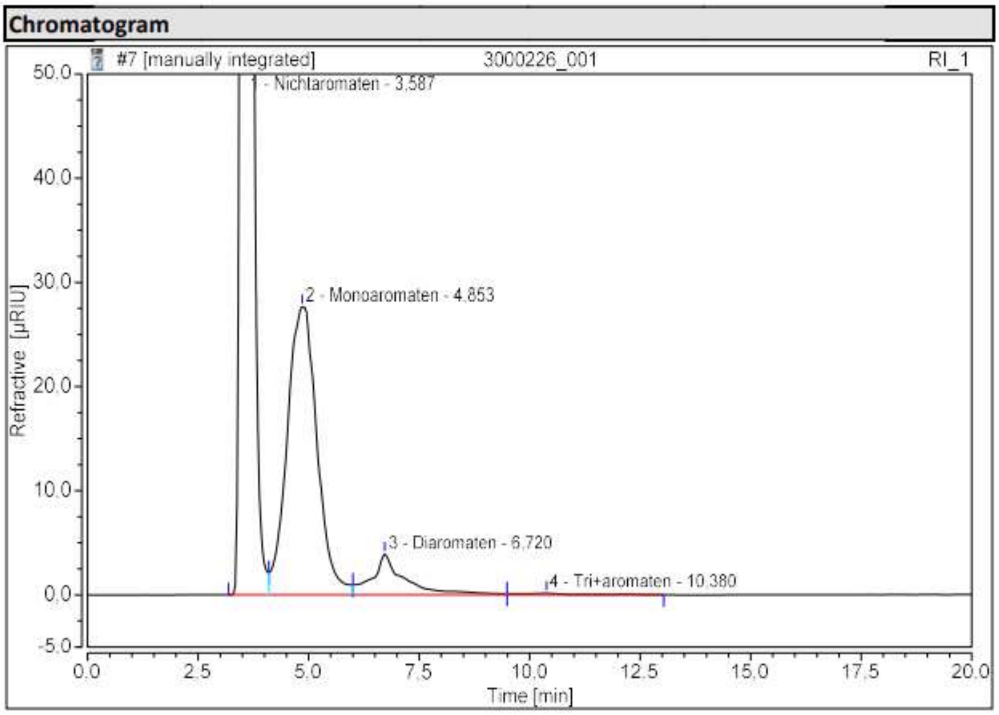

Integration Results

No.	Peak Name	Amount %(m/m)	Retention Time min	Area µRIU*min
1	Nichtaromaten	n.a.	3,587	66,008
2	Monoaromaten	17,28	4,853	21,636
3	Diaromaten	1,44	6,720	3,305
4	Tri+aromaten	0,12	10,380	0,221
	Polyaromaten	*1,56*		
	Gesamtaromaten	*18,84*		

Figure S3*: Chromatogram for the determination of mono-, di-, tri-, and polyaromatic compounds. The analysis is part of the fuel analysis from Table S4.*

3.2 EN590

Biodiesel

Table S5: *Fuel analysis of the biodiesel (rapeseed methyl ester) supplied by ASG-Analytik-Service GmbH (Germany) for the EN 590 investigation.*

Parameter	Method	Result	Unit
Ester content	DIN EN 14103 :2015	98.9	% (m/m)
		8.5	% (m/m)
Density at 15 C	DIN EN ISO 12185 :1997	882.7	kg/m3
Kin. Viscosity at 40 °C	DIN EN ISO 3104 :1999	4.451	mm2/s
Flash point	DIN EN ISO 2719 :2016	156	°C
CFPP	DIN EN 116 :2018	-17	°C

Sulfur	DIN EN ISO 20884 :2011	<5(3.2)	mg/kg
Cetane number	DIN EN 17155 :2018	52.4	-
Ash content (sulphate ash)	ISO 3987 :2010	<0.01	% (m/m)
Water content	DIN EN ISO 12937 :2002	129	mg/kg
Total pollution	DIN EN 12662 :1998	3	mg/kg
Corrosion effect on copper	DIN EN ISO 2160 :1999	1	Korr. Grad
Oxidation stability 110° C	DIN EN 14112 :2016	5.6	h
Acid value	DIN EN 14104 :2003	0.249	mg KOH/g
Iodine value	DIN EN 16300 :2012	108.7	g Iod/100g
PUFA	DIN EN 15779 :2013	<0.60	% (m/m)
Methanol content	DIN EN 14110 :2019	0.04	% (m/m)
Free glycerine content	DIN EN 14105 :2011	0.001	% (m/m)
Monoglyceride content	DIN EN 14105 :2011	0.65	% (m/m)
Diglyceride content	DIN EN 14105 :2011	0.11	% (m/m)
Triglyceride content	DIN EN 14105 :2011	0.02	% (m/m)
Total glycerine content	DIN EN 14105 :2011	0.183	% (m/m)
Alkali metal content (Na+K)	DIN EN 14538 :2006	<1	mg/kg
Alkaline earth metal content (Ca+Mg.)		<1	mg/kg
Phosphorus content	DIN EN 14107 :2003	<4(0.5)	mg/kg
Cloudpoint	DIN EN 23015 :1994	-4	°C

HVO

Table S6: *Fuel analysis of the HVO supplied by ASG-Analytik-Service GmbH (Germany) for the EN 590 investigation.*

Parameter	Method	Result	Unit
Cetane number	DIN EN 17155 : 2018	71.2	-
Density at 15 C	DIN EN ISO 12185 :1997	780.3	kg/m3
Flash point	DIN EN ISO 2719 :2016	78	°C
Kin. Viscosity at 40 °C	DIN EN ISO 3104 :1999	3.028	mm2/s
Volumen at 250 °C	DIN EN ISO 3924 :2019	3.1	% (V/V)
Volume at 350 °C	DIN EN ISO 3924 :2019	106.9	% (V/V)
95 %-Point	DIN EN ISO 3924 :2019	293.1	°C
HFRR	DIN EN ISO 12156-1 :2019	370	µm
FAME content	DIN EN 14078 :2014	<0.1	% (V/V)
Manganese	DIN EN 16136 :2015	<0.5	mg/l
Total aromatic	DIN EN 12916 :2019	<0.1	% (m/m)
Sulfur	DIN EN ISO 20884 :2019	<5(<1)	mg/kg
Coke residue	DIN EN ISO 10370 :2015	<0.10	% (m/m)
Ash content	DIN EN ISO 6425 :2003	<0.001	% (m/m)
Water content	DIN EN ISO 12937 :2002	<30	mg/kg
Total pollution	DIN EN 12662 :2014	<12	mg/kg
Corrosion - Copper	DIN EN ISO 2160 :1999	1	Korr. Grad
Filterable aging residue	DIN EN ISO 12205 :1996	<1	g/m3
Non filterable aging residue	DIN EN ISO 12205 :1996	<1	g/m3
Oxidation stabillity	DIN EN ISO 12205 :1996	<1	g/m3

Oxidation stabillity	DIN EN 15751 :2014	37.5	h
CFPP	DIN EN 116 :2018	-35	°C
Cloud point	DIN EN 23015 :1994	-29	°C
Heating value	DIN 51900-2 :2003 mod.	43913	J/g
Oxygen	DIN 51732 :2014 mod.	<0.5	% (m/m)
Carbon	DIN 51732 :2014 mod.	84.4	% (m/m)
Hydrogen	DIN 51732 :2014 mod.	15.3	% (m/m)
Nitrogen	DIN 51732 :2014 mod.	<0.5	% (m/m)

B0

Table S7: *Fuel analysis of the B0 supplied by ASG-Analytik-Service GmbH (Germany) for the EN 590 investigation.*

Parameter	Method	Result	Unit
Cetane number	DIN EN 17155 :2018	52.6	-
Cetane nindex	DIN EN ISO 4264 :2018	52.4	-
Aromatics, Poly (2+3)	DIN EN 12916 :2022 Verf. A	2.7	% (m/m)
Sulfur	DIN EN ISO 20884 :2019	6.7	mg/kg
Manganese	DIN EN 16576 :2015	<0.50	mg/l
Flash point	DIN EN ISO 2719 :2021	68	°C
Coke residue	DIN EN ISO 10370 :2015	<0.10	% (m/m)
Ash content	DIN EN ISO 6245 :2003	0.002	% (m/m)
Water content	DIN EN ISO 12937 :2002	34	mg/kg
Total pollution	DIN EN 12662 :2014	5	mg/kg
Corrosion - Copper	DIN EN ISO 12205 :1996	1	Korr. Grad
FAME content	DIN EN 14078 :2014	<0.01	% (V/V)
Oxidation stabillity	DIN EN ISO 12205 :1996	<1	g/m³

Oxidation stabillity	DIN EN 16091 :2012	117.01	min
HFRR	DIN EN ISO 12156-1 :2019	440	µm
Kin. Viscosity at 40 °C	ISO 23581 :2020	2.634	mm²/s
Volumen at 250 °C	DIN EN ISO 3924 :2019	38.1	% (V/V)
Volume at 350 °C	DIN EN ISO 3924 :2019	93.3	% (V/V)
95 %-Point	DIN EN ISO 3924 :2019	356.6	°C
CFPP	DIN EN 116 :2018	-23	°C
Density at 15 C	DIN EN ISO 12185 :1997	833.9	kg/m³

3.3 Solketal influence

Biodiesel

***Table S8**: Fuel analysis of the biodiesel (rapeseed methyl ester) supplied by Louis Dreyfus B.V. (Netherlands) for the solketal influence investigation.*

Parameter	Method	Result	Unit
Density at 15°C	DIN EN ISO 12185	882.5	kg / m³
Water content	DIN EN ISO 12937	0.0113	%
Acid value	DIN EN 14104	0.32	mg KOH / g
Oxidation stability, 110°C	DIN EN 14112	11.3	h
Contamination	DIN EN 12662	12	mg / kg
FAME content	DIN EN 14103	98	% m/m)
Iodine value	DIN EN 16300	107.08	g iodine /100 g
Myristic Acid	DIN EN 14103	0.05	% (m/m)
Palmitic Acid	DIN EN 14103	4.39	% (m/m)
Palmitoleic Acid	DIN EN 14103	0.19	% (m/m)
Stearic Acid	DIN EN 14103	1.65	% (m/m)
Oleic Acid	DIN EN 14103	63.45	% (m/m)
Linoleic Acid	DIN EN 14103	17.87	% (m/m)
Linolenic Acid	DIN EN 14103	7.64	% (m/m)
Eicosanoic Acid	DIN EN 14103	0.55	% (m/m)

Eicosenoic acid	DIN EN 14103	1.15	% (m/m)
Docosanoic acid	DIN EN 14103	0.28	% (m/m)
Docosenoic acid	DIN EN 14103	0.17	% (m/m)
Lignoceric acid	DIN EN 14103	0.11	% (m/m)
Nervonic acid	DIN EN 14103	0.1	% (m/m)
Methanol content	DIN EN 14110	0.011	% (m/m)
Monoglycerols	DIN EN 14105	0.506	% (m/m)
Diglycerols	DIN EN 14105	0.112	% (m/m)
Triglycerols	DIN EN 14105	0.099	% (m/m)
Free glycerol	DIN EN 14105	0.004	% (m/m)
Total glycerol	DIN EN 14105	0.159	% (m/m)
Group I metals (Na+K)	DIN 14538	0.4	mg / kg
Group II metals (Mg+Ca)	DIN 14538	0.11	mg / kg
Phosphorus content	DIN EN 14107	1.77	mg / kg
Sulfur content	DIN EN ISO 20846	2.4	mg / kg
CFPP	DIN EN 116	-17	°C
Cloud point	DIN EN ISO 3015	-5.5	°C
Pour point	DIN ISO 3016	-12	°C
Kin. Viscosity 40°C	DIN EN ISO 3104	4.5	mm^2 /s
Flash point	DIN EN ISO 2719	188.5	°C
Carbon residue	DIN EN ISO 10370	0.1	% (m/m)
Cetane number	DIN EN 15195	53.1	-
Sulfated ash	ISO 3987	<0.005	% (m/m)
Copper strip corrosion	DIN EN ISO 2160	1	Korr. Grad rating
PUFA	DIN EN 15779	<0.60	% (m/m)

HVO

Table S9: *Fuel analysis of the HVO supplied by ASG-Analytik-Service GmbH (Germany) for the solketal influence investigation.*

Parameter	Method	Result	Unit
Cetane number	DIN EN 17155 :2018	74.8	-
Density at 15 C	DIN EN ISO 12185 :1997	780.5	kg/m^3
Flash point	DIN EN ISO 2719 :2021	74.5	°C
Kin. Viscosity at 40 °C	DIN EN ISO 3104 :2021	2.973	mm^{2}s
Volumen at 250 °C	DIN EN ISO 3924 :2019	4.5	% (V/V)
Volume at 350 °C	DIN EN ISO 3924 :2019	-	% (V/V)
95 %-Point	DIN EN ISO 3924 :2019	296	°C
HFRR	DIN EN ISO 12156-1 :2019	432	µm
FAME content	DIN EN 14078 :2014	<0.01	% (V/V)
Manganese	DIN EN 16576 :2015	<0.50	mg/l
Total aromatic	DIN EN 12916 :2022 Verf. B	<0.1	% (m/m)
Sulfur	DIN EN ISO 20884 :2022	<5(<1)	mg/kg
Coke residue	DIN EN ISO 10370 :2015	<0.1	% (m/m)
Ash content	DIN EN ISO 6245 :2003	<0.001	% (m/m)
Water content	DIN EN ISO 12937: 2002	<30	mg/kg
Total pollution	DIN EN 12662 :2014	<12	mg/kg
Corrosion - Copper	DIN EN ISO 2160 :1999	1	Korr. Grad
Filterable aging residue	DIN EN ISO 12205 :1996	<1	g/m^3
Non filterable aging residue	DIN EN ISO 12205 :1996	<1	g/m^3
Oxidation stabillity		<1	g/m^3
Oxidation stabillity	DIN EN 15751 :2014	-	h
CFPP	DIN EN 116 :2018	-24	°C
Heating value	DIN 51900-2 :2003 mod.	43.6	MJ/kg
Carbon	DIN 51732 :2014	84.84	% (m/m)
Hydrogen	DIN 51732 :2014	15.41	% (m/m)

Nitrogen	DIN 51732 :2014	<0.5	% (m/m)

B0

Table S10: *Fuel analysis of the B0 supplied by ASG-Analytik-Service GmbH (Germany) for the solketal influence investigation*

Parameter	Method	Result	Unit
Cloudpoint	DIN EN 23015 :1994	-22	°C
Carbon	DIN 51732 :2014	85.9	% (m/m)
Hydrogen	DIN 51732 :2014	14.1	% (m/m)
Nitrogen	DIN 51732 :2014	<0.5	% (m/m)
Oxygen	DIN 51732 :2014 mod.	<0.5	% (m/m)
Cetane number	DIN EN 17155 :2018	65.7	-
Cetane nindex	DIN EN ISO 4264 :2018	61.8	-
Density at 15 C	DIN EN ISO 12185 :1997	821.6	kg/m³
Aromatics, Poly (2+3)	DIN EN 12916 :2019 Verf. A	5.8	% (m/m)
Sulfur	DIN EN ISO 20884 :2022	<5	mg/kg
Flash point	DIN EN ISO 2719 :2021	99.5	°C
Coke residue	DIN EN ISO 10370 :2015	<0.10	% (m/m)
Ash content	DIN EN ISO 6245 :2003	<0.001	% (m/m)
Water content	DIN EN ISO 12937 :2002	76	mg/kg
Total pollution	DIN EN 12662 :2014	<12	mg/kg
Corrosion - Copper	DIN EN ISO 2160 :1999	1	Korr. Grad
FAME content	DIN EN 14078 :2014	<0.1	% (V/V)
Oxidation stabillity	DIN EN ISO 12205 :1996	<1	g/m³
HFRR	DIN EN ISO 12156-1 :2019	600	µm
Kin. Viscosity at 40 °C	DIN EN ISO 3104 :2021	2.639	mm²/s
Volumen at 250 °C	DIN EN ISO 3924 :2019	16.4	% (V/V)
Volume at 350 °C	DIN EN ISO 3924 :2019	93.6	% (V/V)

95 %-Point	DIN EN ISO 3924 :2019	363	°C
CFPP	DIN EN 116 :2018	-12	°C
Manganese	DIN EN 16576 :2015	<0.5	mg/l
Heating value	DIN 51900-2 :2003 mod.	42.8	MJ/kg

3.4 Vapor pressure

Biodiesel

***Table S11**: Fuel analysis of the biodiesel (rapeseed methyl ester) supplied by Louis Dreyfus B.V. (Netherlands) for the vapor pressure investigation.*

Parameter	Method	Result	Unit
Density at 15°C	DIN EN ISO 12185	882.8	kg / m³
Water content	DIN EN ISO 12937	0.0102	%
Acid value	DIN EN 14104	0.36	mg KOH / g
Oxidation stability, 110°C	DIN EN 14112	11.1	h
Contamination	DIN EN 12662	7	mg / kg
FAME content	DIN EN 14103	97.03	% m/m)
Iodine value	DIN EN 16300	106.93	g iodine /100 g
Myristic Acid	DIN EN 14103	0.05	% (m/m)
Palmitic Acid	DIN EN 14103	4.34	% (m/m)
Palmitoleic Acid	DIN EN 14103	0.22	% (m/m)
Stearic Acid	DIN EN 14103	1.6	% (m/m)
Oleic Acid	DIN EN 14103	62	% (m/m)
Linoleic Acid	DIN EN 14103	18.25	% (m/m)
Linolenic Acid	DIN EN 14103	7.8	% (m/m)
Eicosanoic Acid	DIN EN 14103	0.56	% (m/m)
Eicosenoic acid	DIN EN 14103	1.14	% (m/m)
Docosanoic acid	DIN EN 14103	0.29	% (m/m)

Docosenoic acid	DIN EN 14103	0.17	% (m/m)
Lignoceric acid	DIN EN 14103	0.12	% (m/m)
Nervonic acid	DIN EN 14103	0.11	% (m/m)
methanol content	DIN EN 14110	0.004	% (m/m)
Monoglycerols	DIN EN 14105	0.496	% (m/m)
Diglycerols	DIN EN 14105	0.086	% (m/m)
Triglycerols	DIN EN 14105	0.041	% (m/m)
Free glycerol	DIN EN 14105	0.008	% (m/m)
Total glycerol	DIN EN 14105	0.151	% (m/m)
Group I metals (Na+K)	DIN 14538	1.28	mg / kg
Group II metals (Mg+Ca)	DIN 14538	0.06	mg / kg
Phosphorus content	DIN EN 14107	0	mg / kg
Sulfur content	DIN EN ISO 20846	5.67	mg / kg
CFPP	DIN EN 116	-16	°C
Cloud point	DIN EN ISO 3015	-5.3	°C
Pour point	DIN ISO 3016	-12	°C
Kin. Viscosity 40°C	DIN EN ISO 3104	4.5	mm^2 /s
Flash point	DIN EN ISO 2719	188.5	°C
Carbon residue	DIN EN ISO 10370	0.1	% (m/m)
Cetane number	DIN EN 15195	53.1	-
Sulfated ash	ISO 3987	<0.005	% (m/m)
Copper strip corrosion	DIN EN ISO 2160	1	Korr. Grad rating
PUFA	DIN EN 15779	<0.60	% (m/m)

Fossil otto fuel

***Table S12**: Fuel analysis of the fossil otto fuel supplied by ASG-Analytik-Service GmbH (Germany)) for the vapor pressure investigation.*

Parameter	Method	Result	Unit
Research octane number	DIN EN ISO 5164 :2014	98.5	-
Motor octane number	DIN EN ISO 5163 :2014	87.3	-
Lead content	DIN 51461-1 :2018	<0.85	mg/kg
Density (15°C)	DIN EN ISO 12185 :1997	731.3	kg/m³
Sulfur content	DIN EN ISO 20846 :2012	4.8	mg/kg
Manganese (Mn)	DIN EN 16136 :2015	<0.5	mg/l
Oxidation stability	DIN EN ISO 7536 :1996	>360	min
Evaporation residue	DIN EN ISO 6246 :2017	<0.5	mg/100ml
Corrosive effect on copper	DIN EN ISO 2160 :1999	1	Korr. Grad
Olefin content	DIN EN ISO 22854 :2016	9.36	% (V/V)
Aromatic content	DIN EN ISO 22854 :2016	30.48	% (V/V)
Benzene content	DIN EN ISO 22854 :2016	0.32	% (V/V)
Total oxygen otto fuel	DIN EN ISO 22854 :2016	<0.01	% (m/m)
Methanol	DIN EN ISO 22854 :2016	<0.01	% (V/V)
Ethanol	DIN EN ISO 22854 :2016	<0.01	% (V/V)
Isopropanol	DIN EN ISO 22854 :2016	<0.01	% (V/V)
Isobutanol	DIN EN ISO 22854 :2016	<0.01	% (V/V)
tert-Butanol	DIN EN ISO 22854 :2016	<0.01	% (V/V)
Ether (>5C atoms)	DIN EN ISO 22854 :2016	<0.01	% (V/V)
Other oxygen-containing compounds		<0.01	% (V/V)
Vapor pressure DVPE	DIN EN 13016-1 :2018	90.4	kPa
Start of distillation	DIN EN ISO 3405 :2011	27.8	°C
Amount evaporated at 70°C	DIN EN ISO 3405 :2011	35.8	% (V/V)
Amount evaporated at 100°C	DIN EN ISO 3405 :2011	53.4	% (V/V)

Amount evaporated at 150°C	DIN EN ISO 3405 :2011	93.2	% (V/V)
End point of boiling	DIN EN ISO 3405 :2011	181.7	°C
Distillation residue	DIN EN ISO 3405 :2011	1	% (V/V)
